# Deciphering Concentration and Backbone Identity Effects on the Redox Properties of Phthalimide-Based Polymers

**DOI:** 10.1021/acs.chemmater.6c01965

**Published:** 2026-09-14

**Authors:** Khirabdhi Tannaya Mohanty, Sheila Keating, Kha Trinh, Riccardo Alessandri, Daniel P. Tabor, Stuart J. Rowan, Juan J. de Pablo, Jodie L. Lutkenhaus

**Affiliations:** † Artie McFerrin Department of Chemical Engineering, 14736 Texas A&M University, College Station, Texas 77843, United States; ‡ Department of Chemistry, 189300 University of Chicago, Chicago, Illinois 60637, United States; § Pritzker School of Molecular Engineering, 2462 University of Chicago, Chicago, Illinois 60637, United States; ∥ Department of Chemical and Biological Engineering, Tandon School of Engineering, New York University, Brooklyn, New York 11201, United States; ⊥ Department of Chemical Engineering, KU Leuven, Celestijnenlaan 200F, Leuven 3001, Belgium; # Department of Chemistry, 14736 Texas A&M University, College Station, Texas 77840, United States; ∇ Department of Computer Science, Courant Institute of Mathematical Sciences, New York University, New York, New York 10012, United States; ○ Department of Physics, New York University, New York, New York 10012, United States; ◆ Materials Science Division, Argonne National Laboratory, Lemont, Illinois 60439, United States; ¶ Department of Materials Science and Engineering, Texas A&M University, College Station, Texas 77840, United States

## Abstract

Non-conjugated redox-active polymers (NC-RAPs) in the dissolved state are important for future energy applications such as redox flow batteries. However, there remains a knowledge gap in understanding factors that strongly influence the redox kinetics and mechanisms of NC-RAPs. Here, we determine how concentration and backbone structure of phthalimide-based NC-RAPs influence the solution-state electrochemical activity, as described by the apparent diffusion coefficient (*D*_app_) and the homogeneous (*k*_ex,app_) and heterogeneous (*k* ^0^) rate constants. Three phthalimide-containing polymers with epichlorohydrin, methacrylate, and vinylbenzene backbones are compared experimentally and computationally. As polymer concentration increases from the dilute to the semidilute regimes, the diffusion and kinetics slow down. Interestingly, the polymer backbone does not strongly influence the redox kinetics of these phthalimide-based polymers. In total, these results show that, above the overlap concentration, single-file diffusion and interdependent flux of redox species lead to slower diffusion and kinetics. These findings provide mechanistic insights into the future molecular design of NC-RAPs in the dissolved state, enabling energy storage systems such as redox flow batteries with improved kinetics.

## Graphical abstract

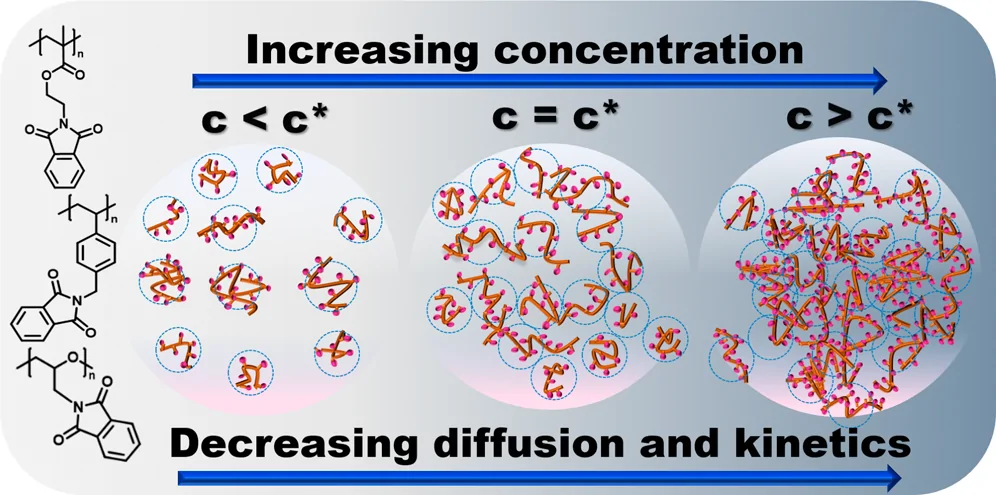

## 1 Introduction

Non-conjugated redox-active polymers (NC-RAPs) are widely explored for energy storage applications such as all-polymer batteries, polymer-air batteries, redox flow batteries and other energy-related-technologies.^1–5^ In their dissolved state, NC-RAPs are potential earth-abundant active materials for redox flow batteries in which charge is stored through the reversible oxidation and reduction of the polymer’s pendant redox groups. NC-RAPs offer a vast synthetic design space, in which different redox units (*e.g.,* viologens,^6,7^ TEMPO radicals,^8–11^ quinones,^12^ phenothiazines,^13–15^ carbazoles,^16–18^ imides^19,20^ ) and backbones (*e.g.,* poly­(ethylene oxide),^9,21,22^ polystyrene,^26–28^ polyacrylates,^23,24^ and poly­(dimethylsiloxane)^25^) are available. However, the influence of molecular effects on the electrochemical kinetics of NC-RAPs is only now emerging. In particular, the effect of solution-level factors such as concentration, chain conformation, and entanglement is still not well understood. These properties are known to strongly influence rheological, mechanical, and other properties,^26–28^ but connections with electrochemical kinetics and mechanisms are scarce.^29–32^ Therefore, a better understanding of these solution-scale effects is needed to more effectively design NC-RAP active materials for next-generation energy storage systems.

N-substituted phthalimides have attracted attention as promising redox units for NC-RAPs, owing to the phthalimide’s chemically reversible redox behavior, energy storage capacity (0.67 Ah L^–1^, 0.05 M^33^), and high electronic coupling.^34^ Phthalimides reduce to corresponding radical anions that are localized on electron-deficient rings at potentials of ∼1.4 V vs SCE.^35–38^ Recently, phthalimide NC-RAPs with three different types of backbones (based on polyepichlorohydrin, polymethacrylate, and polystyrene, Figure 1) were computationally examined for their structural, ionic, and electronic properties in a solid-state redox environment using molecular dynamics and machine learning models by our team.^39^ We showed that phthalimide-based polymers yielded redox kinetic parameters that were three orders of magnitude larger than nitroxide radical-based (2,2,6,6-tetramethylpiperidine-1-oxyl) PTMA; specifically, the phthalimide derived from the polyepichlorohydrin backbone was predicted to yield the fastest kinetics.^39,40^ The question remains as to how these three phthalimide-based polymers perform in the solution state.

**Figure 1:**
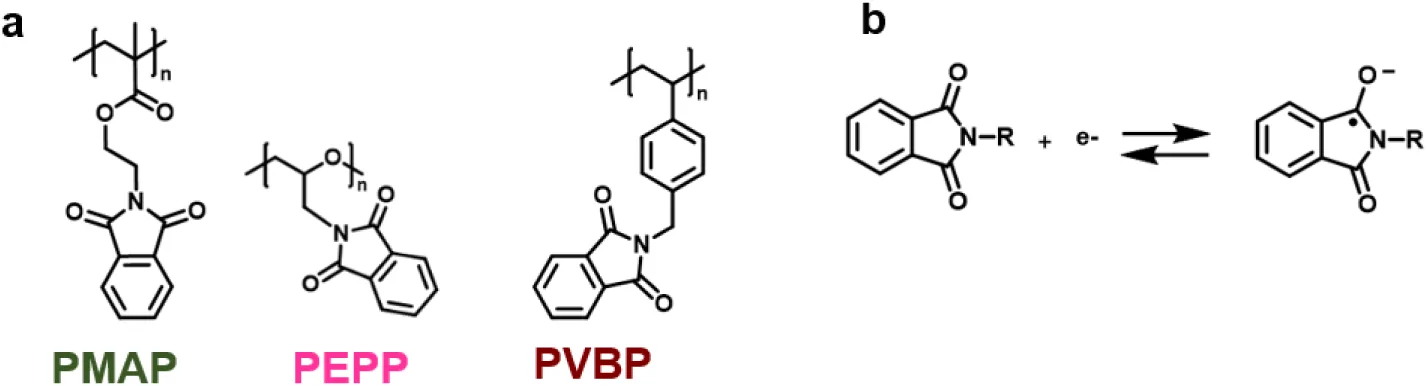
Molecular structures and redox reactions of phthalimide-based NC-RAPs. (a) Molecular structures of (left to right) poly­(*N*-vinylbenzyl)­phthalimide (PVBP), poly­(*N*-methacrylate phthalimide) (PMAP), and poly­(*N*-2,3-epoxypropyl)­phthalimide (PEPP). (b) Phthalimide redox reaction.

In one study of the solution-state electrochemistry of phthalimide-based NC-RAPs from the Schubert group,^41^ a phthalimide copolymer operated in a fully organic redox flow battery yielded 97% voltage efficiency and a capacity of 0.268 Ah L^–1^. Specifically, the copolymer was comprised of a styrenic backbone and comonomers of ethylene glycol side chains for enhanced solubility. Notably, the authors observed substantial reactivity of the reduced phthalimide moiety with certain organic solvents and salts. Other backbones – beyond styrenic ones – have not been widely explored for phthalimides, yet prior studies indicate that backbone-dependent polymer–electrolyte interactions can strongly influence ion transport and capacity.^8,31,42–44^

NC-RAPs, such as the ones described above, transfer charge electrochemically in solution via a three-step charge-transfer mechanism. First, the redox-active species diffuse toward the electrode; then the polymer undergoes molecular/solvent reorganization; last, electron transfers occurs between the NC-RAP and electrode surface, described using the heterogeneous rate constant, k^0^.^1,45^ Within the solution itself, charge transport may occur by hopping from pendant group to pendant group either *intra* or *inter*-molecularly, described using the apparent homogenous (self-exchange) kinetic rate constant (*k*_ex,app_). For the case when the physical diffusion of the polymer is on the order of electron diffusion, an apparent diffusion coefficient is invoked: *D*_app_= *D*_phys_ + *D*_et_.^1,45^

Molecular weight and ionic strength both influence the charge transfer kinetics of NC-RAPs, but the effect of concentration is less explored. Generally, increasing the molecular weight of the NC-RAP leads to decreased diffusion coefficients and decreased k^0^ values because of the polymer’s increased size.^29,30,46^ As for ionic strength, increasing the salt concentration of the electrolyte collapses the polymer chain due to screening effects, which enhances both *D*_phys_ and *D*_et_.^30^ With regard to concentration, Burgess *et al*.^29,30^ explored viologen- and ferrocene-based NC-RAPs below and above the overlap concentration, showing that increasing concentration caused crowding and entanglement that slowed diffusion and electron-transfer kinetics. As for NC-RAPs as grafts on SiO_2_ particles, we showed that increasing the concentration led to increases in D_app_, *k*_ex,app_ and k^0^ due to enhanced interchain charge transport pathways between PTMA chains in solution.^32^ Taken together, the understanding of polymer concentration effects on the redox kinetics is very limited, with only a few polymers being systematically explored,^29,30^ and effect of backbone (keeping the pendant group same) has not yet been examined.

Past theoretical studies have examined the mechanisms of solution-state charge transfer in NC-RAPs as a function of polymer or repeat unit concentration. Bello and Sing predicted that charge diffusion in NC-RAPs increases as the concentration passes from dilute to concentrated regimes, with charge transfer shifting from translational diffusion to interchain hopping.^47^ However, this behavior has not been experimentally validated and is contrary to the observations of Burgess *et al.^29^* ^*–^31^*^ Elsewhere, a similar positive correlation between hole mobility and concentration was experimentally observed in a conjugated polymer solution.^48^ Considering the differing views of theory and experiment in regards to solution-state electron transfer kinetics and polymer concentration, more insight is needed.

In this work, we examine the solution-state charge-transfer kinetics of linear phthalimide-based NC-RAPs of varying polymer concentration and backbone structure. We specifically examine concentrations below and above the overlap concentration, accessing both dilute and semidilute regimes. The backbone chemistry is varied (Figure 1), following our past computational work on the solid-state electrochemistry of polyphthalimides with polymethacrylate, polyepichlorohydrin, and styrene backbones.^39^ These backbones have varying flexibilities and solvent interactions, leading to different overlap concentration and coil sizes. These comparisons are accomplished through electrochemical measurements (cyclic voltammetry and chronoamperometry), along with replica-exchange molecular dynamics simulations that probe single-chain conformations in dilute solutions. Our results reveal that increasing polymer concentration suppresses charge-transfer kinetics, and backbone variations exert minimal influence on the electron-transfer rates. Specifically at high concentrations, crowding of the polymer chains limits charge transfer. These results will serve to guide the future design of redox-active polymer solutions.

## 2 Results and Discussion

### 2.1 Synthesis

Poly­(*N*-vinylbenzylphthalimide) PVBP (*M*_n_ = 13.6 kDa vs polystyrene (PS) standards Đ = 1.6) and poly­(*N*-methacryloxyethyl-phthalimide) PMAP (*M*_n_= 20.4 kDa vs PS std. Đ = 1.5) were obtained via free radical polymerization of their corresponding monomers *N*-vinylbenzyl-phthalimide (VBP) and *N*-methacryloxyethyl-phthalimide (MAP),^41,49^ respectively (see Figures S1 and S2 for nuclear magnetic resonance (NMR) spectra of the polymers). The third polymer, poly­(*N*-2,3-epoxypropyl)­phthalimide (PEPP) (*M*_n_= 41 kDa vs PS std, Đ =3), was synthesized using post-polymerization functionalization of polyepichlorohydrin and was solvent-fractionated to remove shorter polymer chains (see Figure S3). PEPP was characterized as 89% phthalimide-functionalized from ^13^C qNMR (see Figure S4). While the monomers were broadly soluble in organic solvents, the resulting polymers (PVBP, PMAP, and PEPP) exhibited poor solubility in THF, acetone, hexanes, and toluene, sparingly soluble in propylene carbonate and acetonitrile. However, all the polymers exhibited good solubility in chloroform and DMF.

Differential scanning calorimetry measurements showed that the glass transition temperature (*T*_g_) of the dry polymers correlated with backbone flexibility (Figure S5): PVBP (rigid polystyrene backbone) exhibited the highest *T*_g_ (151 °C), whereas PEPP (flexible ether backbone) had the lowest *T*_g_ (93 °C); PMAP was intermediate with a *T*_g_ = 127 °C. All polymers demonstrated good thermal stability, with degradation temperatures (T_95%_) generally exceeding 300 °C (Figure S6).

### 2.2 Overlap Concentration

Measured using rheometry, the overlap concentrations (c*) of PMAP, PVBP, and PEPP in electrolyte (0.1 M TBAPF_6_ in DMF) were 22.5, 30, and 28 mM, respectively. These values are around three times higher than the overlap concentration for PTMA solutions (*M*_n_ ∼ 50 kDa and Đ = 4.62),^32^ and around 0.6 times the overlap concentration for viologen-based RAPs studied at the similar salt concentration.^30^ These differences may be attributed to the different MWs, dispersities, and electrolyte systems.

To assess the c* and radius of gyration (*R*_*g*_) distributions computationally, replica exchange molecular dynamics (REMD) simulations were performed at two degrees of polymerization (N = 10 and N = 20). As shown in Figure 2, the *R*_*g*_ distributions for each polymer and chain length were narrow and well-separated, confirming robust conformational sampling and distinct solution behavior. For all three polymers, *R*_*g*_ consistently increased with chain length, yet the extent of expansion was highly specific to polymer chemistry. PEPP exhibited the most compact structure among the 10-mers, while PMAP showed the largest chain expansion for the 20-mers. These trends reflect the relative molar masses of the polymers’ repeat units, where lower molar masses yield more compact structures. Across all temperatures, the polymer maintained conformational stability throughout the simulations, exhibiting only diffusive fluctuations around a stable mean (see Figures S7–S12). This stability justified averaging values across replicas to obtain ensemble mean and variance from the REMD simulations.

**Figure 2:**
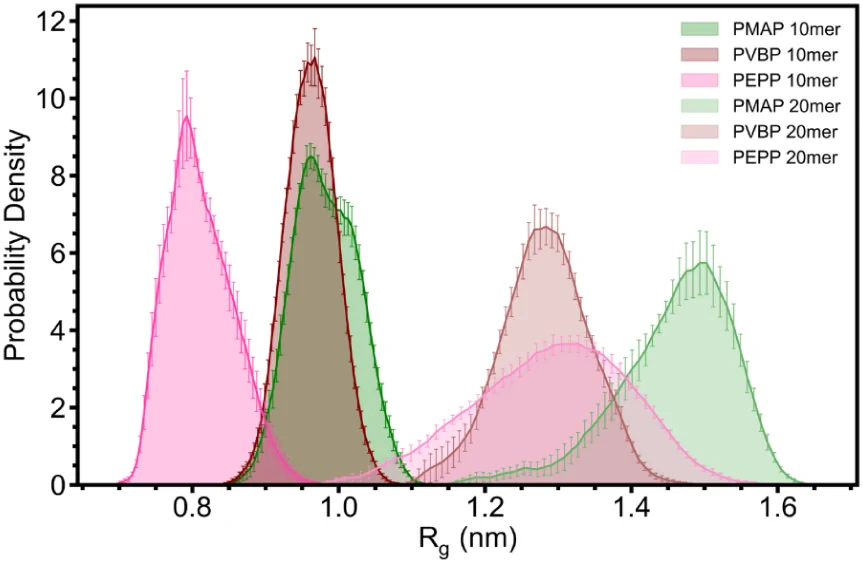
Radius of gyration (*R_g_*) distributions obtained from replica exchange molecular dynamics for PMAP, PVBP, and PEPP polymers at degrees of polymerization N = 10 and N = 20. Each curve represents the probability density of *R_g_*, with error bars indicating the standard error across replicas.

The computed *R*_*g*_ values were next used to determine the scaling exponent ν by fitting the relationship *R*_*g*_ *∼ N* ^*ν*^ using data from N = 10 and N = 20. The resulting fits were then used to extrapolate *R*_*g*_ to experimentally relevant degrees of polymerization, which were inferred from the molecular weights of each polymer under the assumption of a monodisperse distribution (Đ = 1). Values of c* were then calculated using the extrapolated *R*_*g*_ values using eq 1:

1 $$c^{*}=\frac{1}{\frac{4}{3}\pi R_{g}^{3}N_{Av}}$$

The estimated values of c* are 14.4 ± 1.0 mM for PMAP, 54.3 ± 1.7 mM for PVBP, and 10.6 ± 0.7 mM for PEPP, showing limited agreement with experimental results by generally underestimating c* for PMAP and PEPP and overestimating it for PVBP. This discrepancy stems from the simulation’s assumption of ideal, monodisperse chains and the lack of complex inter-chain interactions (entanglement, aggregation) present in real systems, which typically compact the polymer coil and increase the experimental c*. The specific overestimation for PVBP is attributed to intrachain aromatic π–π stacking interactions within its side chains. These strong contacts cause the PVBP coil to be more compact in the simulation than PMAP and PEPP, leading to a calculated c* that is higher than observed experimentally. Therefore, the experimental values of c* were used to prepare solutions for subsequent electrochemical characterization.

### 2.3 Electrochemical Characterization

To compare the electrochemical behavior of phthalimide-based polymers in solution across concentrations from 1/6c* to 3c*, cyclic voltammetry was performed from −2.5 V to −1.5 V vs Ag/Ag^+^ in 0.1 M TBAPF_6_ in DMF, consistent with previous studies.^41^ We first present the results of PEPP in detail, followed by a general comparison with PVBP and PMAP. Figures 3a and 3b show the cyclic voltammograms (CVs) of PEPP at 1/3c* and 3c* for scan rates of 5–200 mV s^–1^, with additional CVs in Figures 3 and S13 for other concentrations. Across all PEPP concentrations, a single pair of redox peaks corresponding to the one-electron transfer of the phthalimide group was observed.^33,41,50^ Reduction generates a phthalimide radical anion that is charge-compensated by a TBA^+^ cation; upon oxidation, the neutral phthalimide unit is recovered. At 1/3c*, the CV indicates quasi-reversible behavior; whereas at 3c*, the CV became broader and more distorted, reflecting increased irreversibility with increasing concentration. We next examined the relationship of the CV’s peak current (*i*_p_) with scan rate (*v*) in the oxidation step. A plot of *i*_p_ vs *v* ^*1/2*^ was linear for 1/3c* (Figure 3c, pink squares) which is expected for a diffusion-limited redox reaction.^51^ For 3c* (Figure 3c, blue circles), *i*_p_ was linear with *v* ^*1/2*^ up to 20 mV/s and plateaued at higher scan rates, indicative of a limiting current density. The reversibility of the PEPP redox reaction was assessed by plotting the peak separation against the scan rate (Δ*E*_p_ vs *v*, Figure 3d). At 1/3c*, Δ*E*_p_ increased from 119 to 235 mV, as the scan rate increased from 5 to 200 mV/s. Whereas, at 3c*, Δ*E*_p_ was significantly greater, increasing from 149 to 440 mV as scan rates increased. Taken together, the redox reaction of PEPP in solution becomes more irreversible in nature as the concentration of PEPP increases.

**Figure 3:**
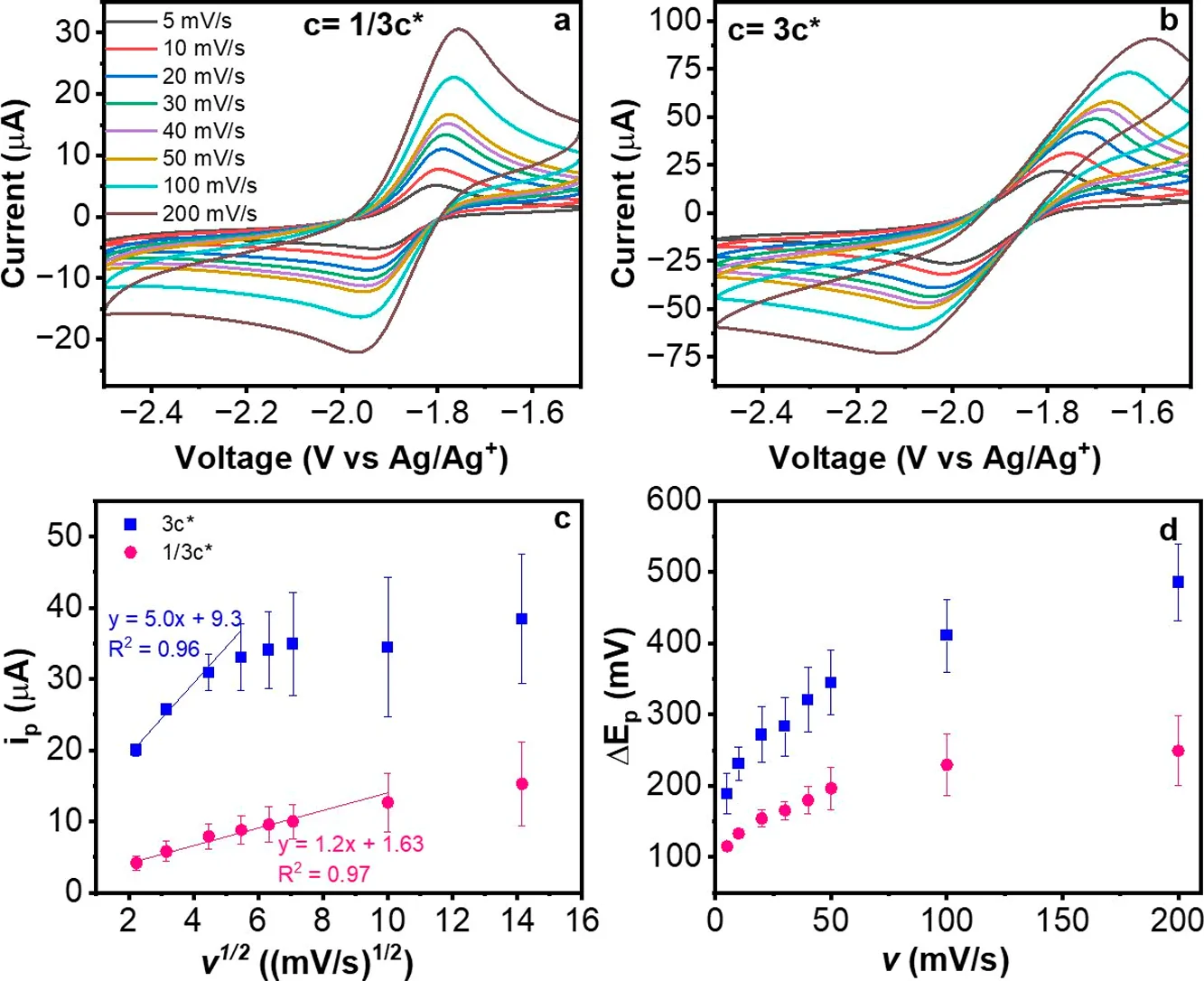
Cyclic voltammogram analysis of PEPP in 0.1 M TBAPF_6_ in DMF. Cyclic voltammograms for concentrations of (a) 1/3c* and (b) 3c*, along with resulting comparisons of (c) peak oxidation current vs sq.rt. scan rate and (d) Δ*E*_p_ vs scan rate. The legends in (a) and (c) apply to (b) and (d), respectively. The working electrode was a glassy carbon electrode (GCE), the reference electrode was Ag/AgNO_3_, and the counter electrode was platinum (Pt) wire. The scan rate varied from 5 to 200 mV/s.

The full collection of CVs for concentrations from 1/6c* to 3c* are displayed in Figure S13. In the dilute region (1/6c*–1/2c*), quasi-reversible CVs were observed, with slight distortion at high scan rates (100–200 mV/s). Furthermore, in the more dilute regime (Figure S13c, 1/4*c* ^*^), a distinct secondary cathodic feature/shoulder is discernible at −2.2 V. At c = c* and in the semidilute region (2c*, 3c*) the cathodic wave extends further from *E*_1/2_ than the corresponding anodic wave, resulting in an asymmetrical, broadened cathodic peak profile (*e.g.,* Figure S13d, S13e and S13f) with increasing Δ*E*_p_. This suggests that the redox reaction becomes more irreversible, which indicates non-Nernstian behavior possibly associated with the adsorption of PEPP at the GCE surface. To investigate this possibility, CVs were recorded on GCEs previously exposed to PEPP solutions in blank electrolyte (0.1 M TBAPF_6_, no polymer; see Figure S14), revealing small redox peaks that grew with prior testing concentration, confirming minor sorption at 1/2c*, and significant sorption at 3c*. This type of sorption behavior was also observed in a solution-state study of polyviologens by Burgess *et al*.^30^ Therefore, we attribute the broadening of the reduction peak at 3c* to the sorption of PEPP. Given that the oxidation peak does not show similar broadening, neutral PEPP likely sorbs to the GCE and dissolves into the electrolyte upon reduction to the radical anion form (see Figure S15). Additionally, we observe an irreversible reduction peak around −2.1 V for the GCE that had been used in the tests conducted at bulk concentrations c*, 2c*, and 3c*. This can be due to PEPP’s broad molecular weight distribution (PDI = 3.3, Figure S4) resulting in non-uniform redox potentials and multi-species responses at higher concentrations.

To further understand the effects of concentration, CVs taken at one scan rate (20 mV/s) were plotted together for solutions containing varying PEPP concentrations, Figure 4. As expected, the peak oxidation and reduction currents increased with increasing PEPP concentration, *i*_p_ ∝ [PEPP] (see Figure 4a and 4b). Δ*E*_p_ and the half-wave potential, *E*_1/2_, were largely within error for the different concentrations, Figure 4c, d.

**Figure 4:**
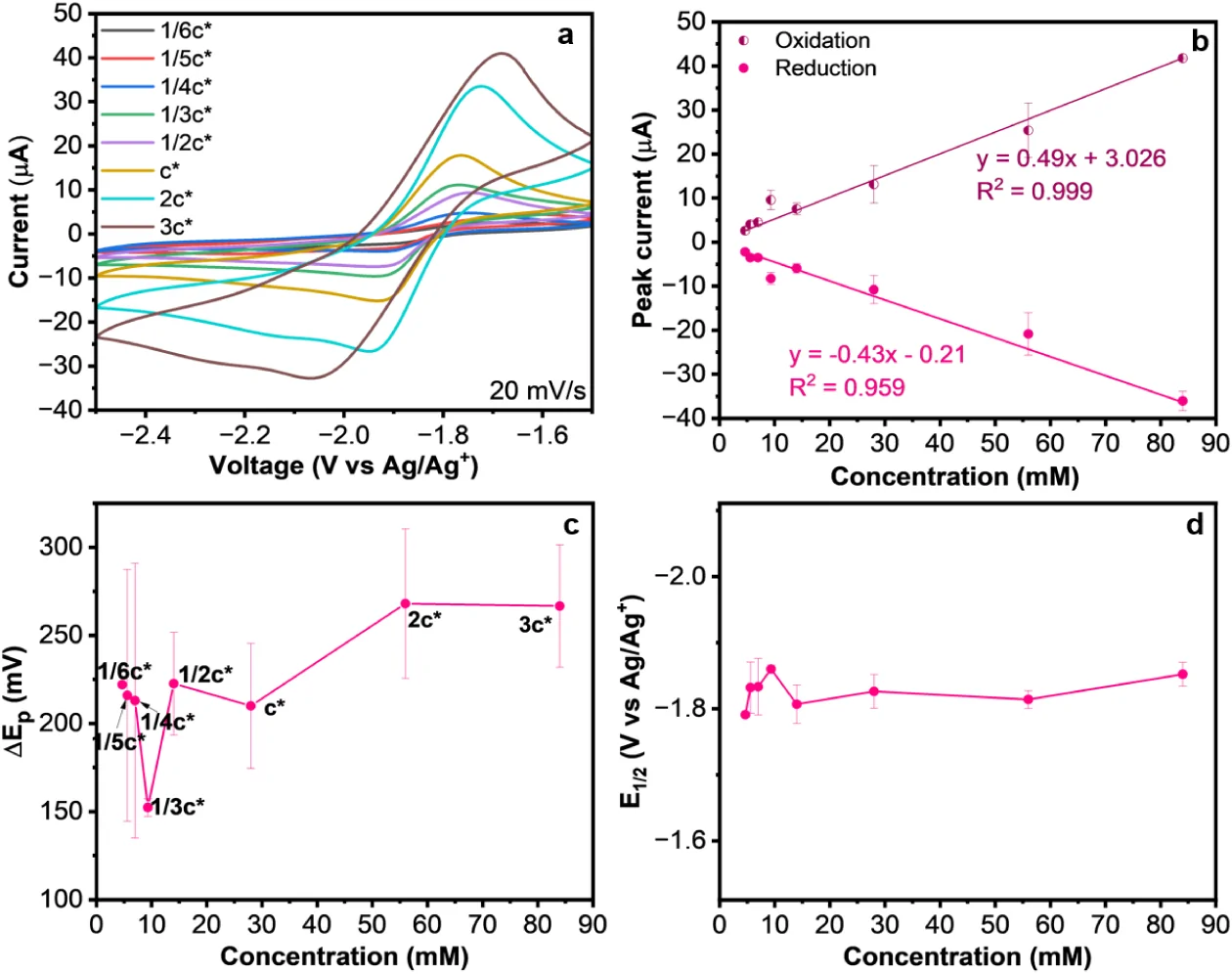
CV trends for different concentrations of PEPP in 0.1 M TBAPF_6_. (a) CVs, (b) Peak currents, (c) peak separations, and (d) half-wave potentials for PEPP concentrations ranging from 1/6c* to 3c*. The working electrode was a GCE, the reference electrode was Ag/AgNO_3_, and the counter electrode was Pt wire. The scan rate was 20 mV/s.

The redox behavior of PMAP and PVBP under similar conditions was also investigated (Figures S15–S21), allowing us to compare with PEPP. Figure 5 shows CVs of each polymer at 5 mV/s for concentrations of 1/6c*, c*, and 3c* for which the currents are normalized against the peak reduction current. At 1/6c* (Figure 5a), all polymer solutions exhibited quasi-reversible behavior, with irreversibility increasing for c* and 3c*, as indicated by the broadened redox peaks and increased peak separation (Figure 5b and 5c). Furthermore, the ratio of the oxidation and reduction peak currents was less than one for all cases; this can be attributed to adsorption and stripping of the polymer, as discussed earlier for PEPP. In comparison with other strongly adsorbing systems,^30,46^ surface adsorption in our system was relatively weak. Instead, diffusion-controlled mass transport remains the primary driver of the electrochemical behavior. To compare across PMAP, PEPP, and PVBP, the normalized oxidation currents and peak separations showed similar concentration-dependent behavior. PVBP and PMAP maintained an *E*_1/2_ of approximately −1.85 V to −1.95 V vs Ag/Ag^+^, consistent with previous studies.^44^ However, the *E*_1/2_ of PEPP was approximately 0.10 V higher than that of PVBP and PMAP at each concentration. This indicates that the higher molecular weight and/or the dispersity of PEPP may shift the redox process to slightly more positive potentials

**Figure 5:**
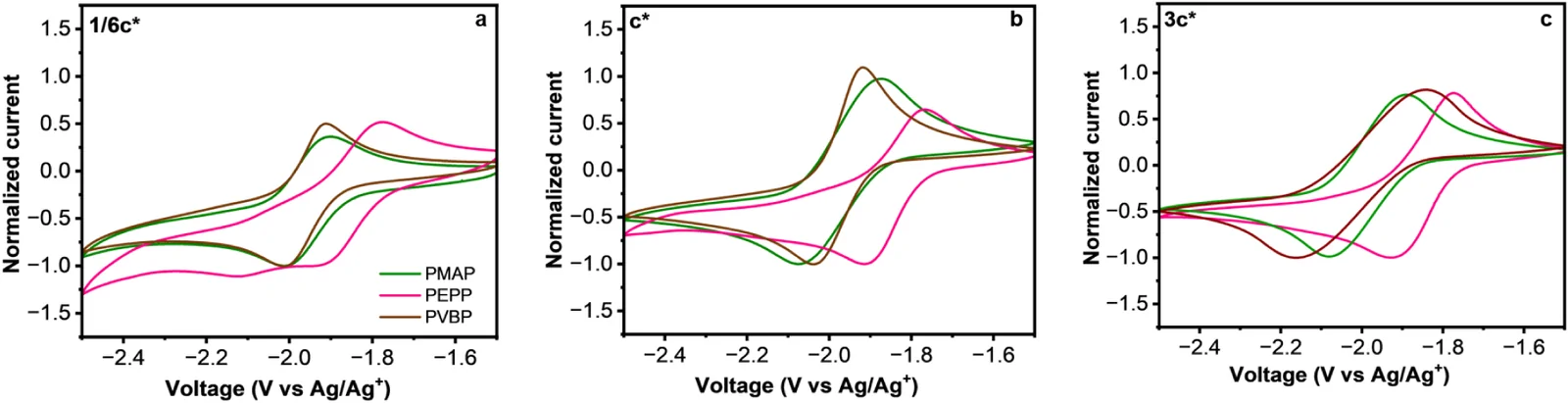
Cyclic voltammograms of PEPP, PMAP and PVBP in 0.1 M TBAPF_6_ in DMF at respective concentrations of (a) 1/6c*, (b) c*, and (c) 3c* at 5 mV/s.

We next examined the apparent diffusion coefficients of the three polymers’ redox reaction taken at varying concentrations as determined using chronocoulometry, Table S1. An example of an Anson plot extracted from the chronocoulometry of a PEPP solution is shown in Figure S22. To simplify the understanding, we show only the D_app_ values for the reduction of PEPP solutions of varying concentration in Figure 6a, whereas those for oxidation are shown in Figure S23. In the dilute regime (1/6c* to 1/3c*), D_app_ during reduction was around 2 × 10^–7^ cm^2^/s. At 1/2c* *D*_*app*_ decreased to 7.4 × 10^–8^ cm^2^ s^–1^. At higher concentrations, D_app_ further decreased to about 4.5 × 10^–8^ cm^2^ s^–1^. Comparatively, dynamic light scattering (DLS) experiments (Figure S24) showed no significant change in PEPP’s physical diffusion coefficient (about 4.8 × 10^–7^ cm^2^/s) over changes in concentration.

**Figure 6:**
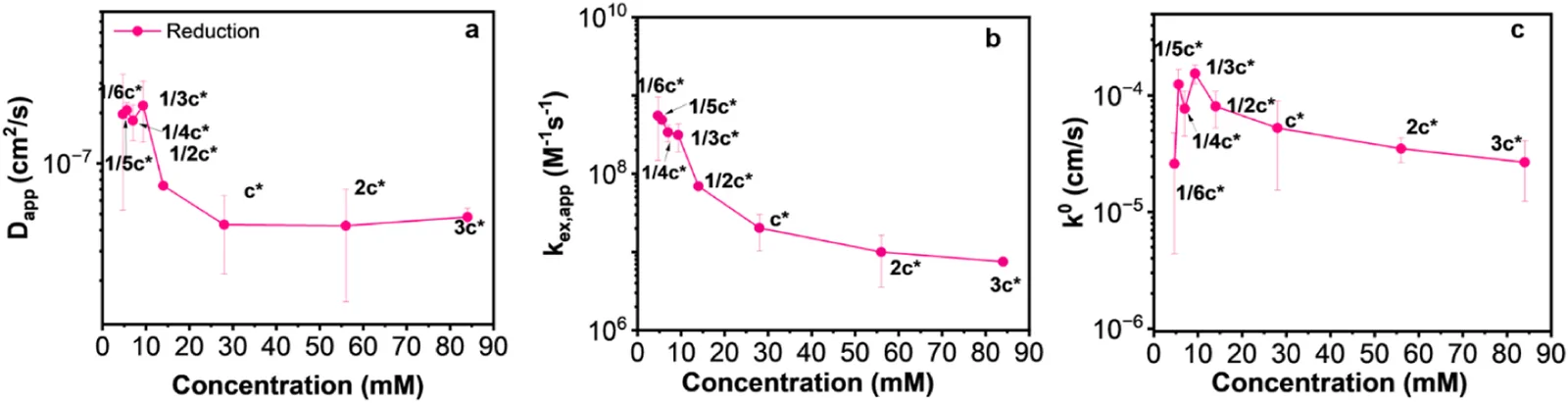
Diffusion and kinetic parameters for the reduction of PEPP with different solution concentrations. (a) Apparent diffusion coefficient (*D_app_*), (b) homogeneous rate constant (*k_ex,app_*), and (c) heterogeneous rate constant (*k* ^0^).

Using the obtained values of D_app_ we applied a diffusion cooperative model, along with the Dahms-Ruff equation and Nicholson method, to calculate the homogenous self-exchange rate constant (*k*_*ex,app*_) and the heterogenous rate constant (*k* ^0^),^30,31^ (Table S1 and Figure 6b and c). For concentrations from 1/6c* to 1/3c*, the value of *k*_*ex,app*_ was approximately 4.2 × 10^8^ M^–1^ s^–1^ for reduction; as concentration further increased, *k*_*ex,app*_ continually decreased down to 7.5 × 10^6^ M^–1^ s^–1^ at 3c*. This ∼56-fold decrease in *k*_*ex,app*_ is a manifestation of the reduced diffusion caused by chain crowding.^52,53^ This suggests that, although the concentration of redox sites is higher, the mobility of those sites is reduced enough so as to yield an overall reduction in self-exchange charge transfer. Separately, the *k* ^0^ values for PEPP (Figure 6c) were generally around 4 × 10^–5^ cm/s for reduction with a slight decrease with increasing concentration. We expect that *k* ^0^ is less sensitive to concentration because *k* ^0^ represents a surface reaction (as opposed to a volumetric one such as for *k*_*ex,app*_). The trends of *D*_*app*_, *k*_*ex,app*_, and *k* ^0^ during oxidation of PEPP were similar to reduction and shown in Figure S23.

We also applied a similar methodology to solutions of PVBP and PMAP of varying concentration (Figure 7, Figures S24–S26). For simplicity and clarity, Figure 7 presents only the diffusion-kinetic behavior for the reduction of PEPP, PMAP, and PVBP. In Figure 7a, PVBP and PEPP both exhibit decreases in *D*_*app*_ with increasing concentration, with PEPP’s decrease being more extreme than that of PVBP. Meanwhile, the value of *D*_*app*_ for PMAP remained largely constant (within experimental error) regardless of concentration. As for the values of *k*_*ex,app*_ and *k* ^0^ for PVBP and PMAP, the trend followed that of PEPP, decreasing with increasing concentration during reduction, Figure 7b and 7c. Specifically, the values of *k*_*ex,app*_ decreased 10-fold from 1/4c* to 3c* for PMAP, PVBP, and PEPP. Likewise, the trends in *k* ^0^ (Figure 7c) were largely similar among the three polymers, with a 4-fold decrease as concentration increased. Given the comparable values of the solution-state diffusion-kinetic parameters across the three polymers studied here, we conclude that, for these systems, the concentration of the active units matters more than the specific identity of the polymer backbone. This conclusion contrasts with our previous simulations of solid-state redox-active polyphthalimides for which PEPP exhibited faster kinetics than PVBP or PMAP,^39^ which suggests that backbone identity could have stronger influence in the solid-state than in the solution state.

**Figure 7:**
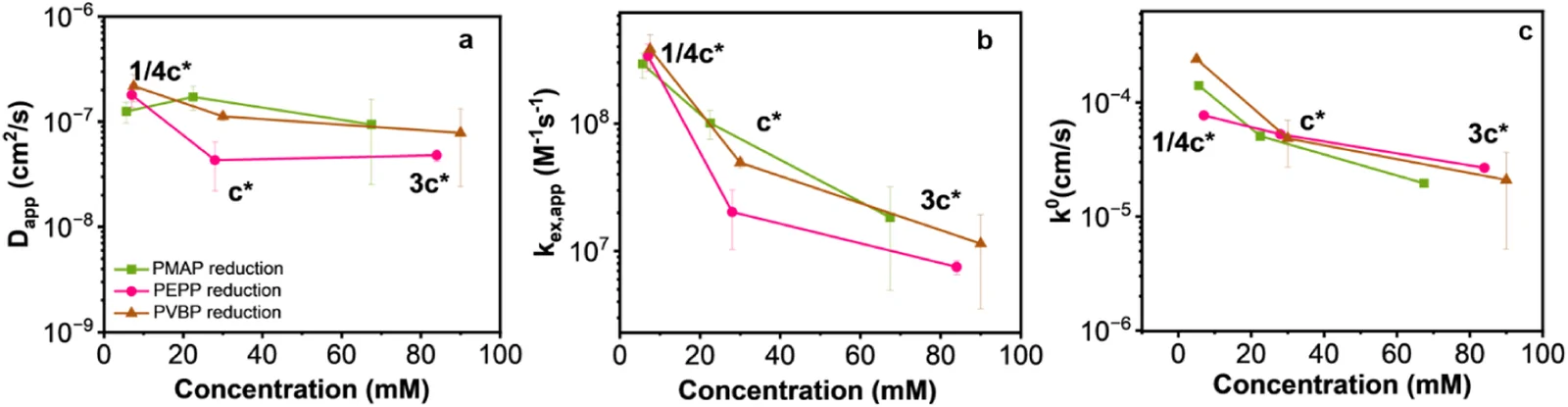
Diffusion and kinetic parameters for the reduction of PMAP, PEPP, and PVBP from chronocoulometry at 1/4c*, c*, and 3c*. (a) *D_app_*, (b) *k_ex,app_*, and (c) *k* ^0^. Only three concentrations are displayed for clarity.

Our observed decrease in *D*_*app*_ with increasing concentration also contrasts with the computational results of Bello and Sing for solution-state NC-RAPs,^47^ where increasing the polymer concentration into the semidilute regime is predicted to promote interchain electron hopping between crowded chains, leading to higher *D*_*app*_ values. Contrastingly, Burgess *et al.^30^* showed that, in solution-state polyviologen redox systems, the diffusion coefficients decreased with increasing NC-RAP concentration, similar to our results for polyphthalimides. In their study, at concentrations higher than the overlap concentration, enhanced polymer sorption at the electrode formed an interfacial polymer layer that dominated the electrochemical response. As a result, redox kinetics were governed by diffusion within this sorbed layer rather than by bulk transport, leading to mass-transport limitations and an apparent reduction in diffusion. It has been shown that a decrease in *D*_*app*_ is observed for small molecule redox moieties diffusing through polymer-coated electrodes.^52,53^ The authors discussed that, at higher concentrations of the redox moiety, diffusion pathways become restricted due to crowding of the polymer layer on the electrode, leading to a single-file diffusion regime in which the fluxes of individual redox couples become mutually dependent.^53^ By analogy to their observations, we propose that our system exhibits similar diffusion restrictions at elevated redox species concentrations. As shown in Figure 8, in the dilute regime (c < c*), polymer chains have unrestricted pathways for diffusion through the electrolyte, providing sufficient space for electron hopping via chain–chain collisions and translational motion, as described by Bello and Sing.^47^ At concentrations approaching and exceeding c*, polymer chains become crowded and rely on neighboring redox species to facilitate charge diffusion within the bulk electrolyte, giving rise to single-file diffusion behavior. Yet, at high concentrations, we observed polymer adsorption onto the electrode surface. This adsorption likely arises from the combined effects of polymer–electrode affinity and local concentration gradients, which constrain the motion of adsorbed chains. As a result, the fluxes of redox species become interdependent, because the motion of one chain can block or facilitate the transport of neighboring species, leading to correlated or “coupled” flux behavior that impacts overall electron transport and diffusion kinetics.

**Figure 8:**
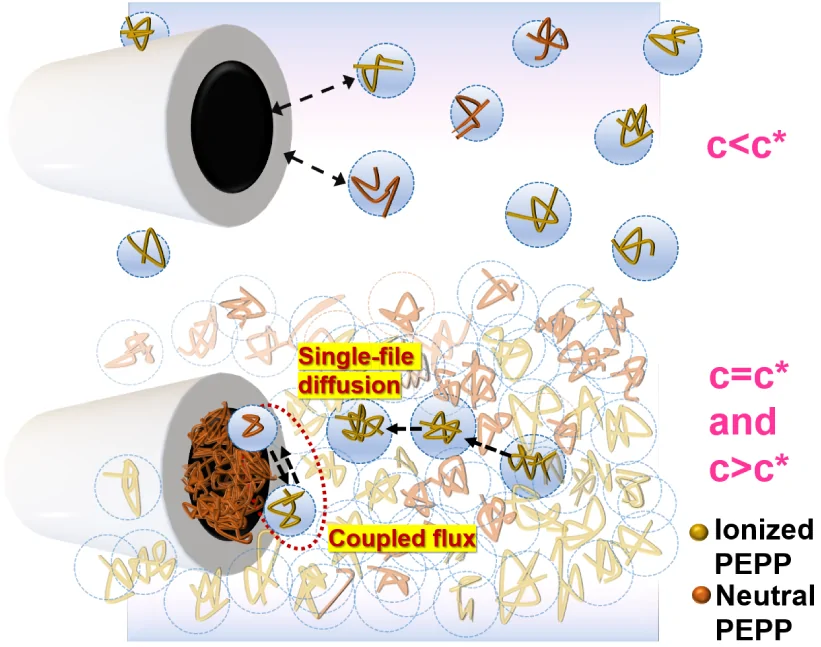
Unrestricted and restricted diffusion in NC-RAP solutions. (Top) Below c*, polymer chain diffusion is unrestricted, resulting in faster apparent diffusion-kinetic parameters. (Bottom) Above c*, polymer chain diffusion is restricted and exhibits single-file diffusion with coupled transport.

Our team’s prior simulations projected a *D*_*app*_ value of ∼10^–6^ cm^2^/s for polyphthalimides as solid electrodes infiltrated with electrolyte.^39^ Here, in comparison, we experimentally estimated a *D*_*app*_ of ∼10^–8^ cm^2^/s for dilute polyphthalimide solutions, which, as suggested by ref 45, projects to a solid-state value of 10^–11^–10^–10^ cm^2^/s, which is not in agreement with our solid-state simulations in the prior study. Such differences arise due to disparities in polymer chain length, electrolyte concentration and solvent, and the measurement time scale. Most notably, electron hopping contributions are computed here to be three orders of magnitude lower than in ref 39 (∼10^–9^ cm^2^/s here *vs* ∼10^–6^ cm^2^/s in ref 39), making this the largest single contributor to the discrepancy. As described in the next paragraph, this arises from considerably larger estimated reorganization energies due to the different solvent environment (see Figure S29). Importantly, these reorganization energies are computed using the same methodology as in ref 39, so the difference reflects the change in solvent environment rather than a change in computational approach. It remains to be seen whether the difference in environment (a solution of DMF here vs a solvent-swollen polymer matrix in ref 39) is realistically accounted for by the very simple model that we use to compute the reorganization energy.

To bridge the gap between macroscopic experimental data and theoretical kinetic behavior, we performed molecular dynamics (MD) simulations to probe the concentration-dependent transport mechanisms at the molecular scale. From the concentration-series simulations, the physical diffusion coefficients were obtained directly from the monomer-center-of-mass mean squared displacement (MSD). Under dilute conditions, *D*_*phys*_ was (1.25 ± 0.08) × 10^–6^ cm^2^/s (PMAP), (1.56 ± 0.10) × 10^–6^ cm^2^/s (PVBP), and (2.10 ± 0.13) × 10^–6^ cm^2^/s (PEPP). Under semidilute conditions, *D*_*phys*_ decreased to (1.14 ± 0.07) × 10^–6^ cm^2^/s (PMAP), (1.12 ± 0.08) × 10^–6^ cm^2^/s (PVBP), and (1.52 ± 0.09) × 10^–6^ cm^2^/s (PEPP). In all cases, we noted that the hopping contribution was negligible in the solution state (*D*_*e*_ ≪ *D*_*phys*_) due to the large reorganization energies (see Section S1 and Figures S28, S29), so we took the approximation that *D*_*app*_ ≈ *D*_*phys*_ and reported *D*_*app*_ from the molecular dynamics trajectories.

The observed decrease in *D*_*app*_ when transitioning from the dilute to the semidilute regime is qualitatively consistent with our experimental findings, confirming the expected shift in dynamics due to increased crowding (single-file diffusion and coupled flux, *vide infra*). However, a quantitative discrepancy remains: the experimental values of *D*_*app*_ are approximately one order of magnitude lower than those obtained from simulations. This could be attributed to the short length of the polymer chains in the simulation and also to the differing time scales at which the measurements are performed (5 s for experiments and 0.2 ns for simulations). Interestingly, our current solution-state simulations yielded *D_app_* values within the same order of magnitude as our previous solid-state simulations, which utilized 30-mer chains. This numerical agreement is noteworthy considering the disparate conditions—including variations in chain length, electrolyte composition, and temperature—and suggests that the underlying transport physics remains relatively consistent across these different polymeric environments.

The overall *D_app_* values for the phthalimide-based polymers in this study were comparable to those of other solution-state polymers,^10,29–31,54^ (see Tables S1 , S2, S3 and S4) validating their potential as active materials for polymer-based batteries. The *k*_ex,app_ values of phthalimide-based polymers (10^6^–10^8^ M^–1^ s^–1^) were in the upper limit of the range in the diffusion cooperative model proposed by Sato *et al.* for redox-active dissolved polymers, which was 10^4^–10^8^ M^–1^ s^–1^,^45^ suggesting fast electron self -exchange rate. The *k*_ex,app_ values are further supported by their comparability to *k*_ex,app_ values in high-performance viologen-based polymers reported by Burgess *et al.*,^29–31^ (Table S4). Additionally, the k^0^ values of phthalimide-based polymers studied here fall within the established range for fast charging redox active polymers, as predicted by Sato *et al.* ^54^ Collectively, these metrics demonstrate that phthalimide-based polymers possess charge-transport kinetics that are among the higher range for redox-active polymers in the literature.

## 3 Conclusion

We synthesized phthalimide-based polymers with three different backbones using free radical polymerization (PMAP and PVBP) and post-polymerization functionalization of PEPP. The redox kinetics of the three phthalimide-based polymers were evaluated at different concentrations relative to the overlap concentration using a diffusion-cooperative model to extract the concentration-dependence of *D*_app_, *k*_ex,app_, and *k* ^0^. For PVBP, PMAP, and PEPP, both *D*_app_ and *k*_ex,app_ decreased with increasing concentration into the semidilute regime in which enhanced chain overlap crowding impedes polymer mobility and electron hopping efficiency. In contrast, *k* ^0^ remained less sensitive to concentration (∼10^–5^ cm s^–1^), indicating that the intrinsic surface electron-transfer kinetics. Across these three systems, comparable overall transport kinetics were observed (*D*_app_ ∼ 10^–8^ cm^2^ s^–1^ and *k*_ex,app_ ∼ 10^7^ M^–1^ s^–1^), although the experimental *D_app_* values were approximately two orders of magnitude lower than simulation predictions, likely reflecting idealized assumptions in the computational model.

Overall, the kinetic parameters estimated for this system are commensurate with reported values of high-performance TEMPO-based polymers (*D*_app_ ∼ 10^–6^–10^–8^ cm^2^ s^–1^ and *k* ^0^ ∼ 10^–3^–10^–5^ cm s^–1^) and viologen-based polymers (*D*_app_ ∼ 10^–7^–10^–8^ cm^2^ s^–1^, *k*_ex,app_ ∼ 10^6^–10^8^ M^–1^ s^–1^ and *k* ^0^ ∼ 10^–3^–10^–5^ cm s^–1^) in solution.^10,29–31,54^ These results, combined with their favorable self-exchange rates, position phthalimide-based materials as highly promising anodic candidates for organic batteries.

We conclude that the solution-state charge transfer kinetics of the phthalimide-based polymers are primarily concentration-dependent rather than determined by the polymer backbone structure itself. For future applications in redox flow batteries, scaling these systems will require pushing active species concentrations to practical concentrations that far exceed the overlap concentration (≥2 M) without severe viscosity penalties and, as shown here, decreased *D*_app_ and *k*_ex,app_ values. To further improve these redox kinetics, future structural modifications—such as the incorporation of an alkyl chain or an ionic-charged moiety to enhance chain mobility or increase favorable interchain interactions—are proposed. Another route for improvement is to include NC-RAP-grafted particles, such as in ref 32, which promote inter-chain electron transfer. In future work we are also exploring phthalimides with electron-withdrawing groups to modulate the redox potential and further improve performance. Additionally, we are investigating crosslinked phthalimide polymers as solid-state electrodes for static batteries.

## 4 Experimental Section

### 4.1 Materials

Potassium phthalimide (98%), 4-vinylbenzyl chloride (95%), *N*-(2-hydroxyethyl)­phthalimide (99%), 4-(dimethylamino)­pyridine (DMAP) (99%), and 2,2′-azobis­(2-methylpropionitrile) (AIBN) (98%) (recrystallized from methanol before use) were purchased from MilliporeSigma. Methacrylic acid (stabilized with MEHQ, 99%) was purchased from TCI Chemicals. Tetrabutylammonium bromide (TBAB) (99%) was purchased from Oakwood Chemicals. Polyepichlorohydrin (PECH, Mw = 700,000 g mol^–1^) was purchased from Scientific Polymer Products. Chromium­(III) acetylacetonate (Cr­(acac)_3_, 99%) was purchased from Acros. Deuterated chloroform (CDCl_3_, (D, 99.96%) +0.05% v/v TMS) was purchased from Cambridge Isotope Laboratories. 1-(3-(Dimethylamino)­propyl)-3-ethylcarbodiimide hydrochloride (EDC·HCl, 97%) was purchased from Combi-Blocks.

For electrolyte preparation, tetrabutylammonium hexafluorophosphate (TBAPF_6_) was purchased from Thermo Scientific and non-anhydrous N,N-dimethylformamide (99.8% DMF) was purchased from Sigma-Aldrich. The reference electrode Ag/AgNO_3_ (CH Instruments) was filled with 0.01M AgNO_3_ salt that was purchased from Fisher Scientific. The counter electrode, 0.5 mm dia. platinum wire (99.9% trace metals basis) was purchased from Milipore Sigma. All chemicals were used without further purification except where noted.

### 4.2 Synthesis of Phthalimide Polymers

VBP and MAP monomers were prepared according to previous studies.^41,49^

#### 4.2.1 General Procedure for Polymerization

Monomer and AIBN initiator were mixed with solvent in a 40 mL vial equipped with stir bar and septa cap, then degassed for 1 h using argon before being sealed and heated to 70 °C. The reaction was cut off by exposing to air, then precipitating crude solution in a swirling acetone bath, and filtering out the liquid and isolating the solid polymer. The polymer was then dried in a vacuum oven at ∼150 °C overnight before characterizing.

PVBP: Using DMF as solvent and 200:1 [M]:[I] ratio, polymerization was taken to 47% conversion. ^1^H NMR: See Figure S1.

PMAP: Using MeTHF as solvent and 200:1 [M]:[I] ratio, polymerization was taken to 30% conversion. ^1^H NMR: See Figure S2.

#### 4.2.2 PEPP Synthesis via Post-Polymerization Functionalization of PECH

Commercial poly­(epichlorohydrin) PECH (1 g, 10.8 mmol) was pre-dissolved in DMF in 100 mL in a round bottom flask equipped with stir bar and septa at 140 °C before potassium phthalimide (2.22 g, 12.0 mmol) was added. The polymer was allowed to react for 24 h. At elevated temperatures, the elimination reaction competes with the substitution reaction, leading to a decrease in molecular weight of the product compared to the starting material. The polymer was purified by precipitating the crude mixture into a stirred DI water bath, then filtering out the polymer. This was repeated once by dissolving the crude polymer in chloroform and reprecipitating to remove unreacted phthalimide salt. A batch of PEPP was synthesized, purified, and then fractionated by mixing PEPP with hot THF and decanting the supernatant, leaving PEPP behind and removing lower molecular weight species (see Figure S4). The product was 89% phthalimide-functionalized. PVBP, PMAP, and PEPP were insoluble in THF, acetone, hexanes and toluene and were sparingly soluble in propylene carbonate and acetonitrile. PMAP, PVBP, and PEPP were highly soluble in chloroform and DMF.

### 4.3 Polymer Characterization

#### 4.3.1 Nuclear Magnetic Resonance (NMR) Spectroscopy

NMR spectroscopy was acquired on a fully automatic 400 MHz Bruker Avance-III-HD nanobay spectrometer equipped with a BBFO iProbe and a 60-sample SampleCasePlus autosampler using Topspin 3.6.5 and a 500 MHz Bruker Neo spectrometer equipped with a BBFO iProbe (low-F background) and a 24-sample SampleCase autosampler using Topspin 4.4.1 at the University of Chicago. For quantitative ^13^C NMR spectroscopy, PEPP was dissolved in CDCl_3_ at ∼1.2 M, with 0.1 M Cr­(acac)_3_.

#### 4.3.2 Dynamic Light Scattering (DLS)

DLS was performed using a Wyatt Mobius Dynamic DLS at the Soft Matter Characterization Facility at the University of Chicago.

#### 4.3.3 Gel Permeation Chromatography (GPC)

Molecular weight was measured using a Tosoh EcoSEC GPC equipped with SuperAW3000 + SuperAW4000 + guard column at the University of Chicago. Polymers were eluted with DMF and 0.01 M LiBr at 50 °C.

#### 4.3.4 Differential Scanning Calorimetry (DSC)

DSC was performed using a TA Instruments Discovery 2500 differential scanning calorimeter in the Soft Matter Characterization Facility at the University of Chicago. Samples were prepared in aluminum hermetic pans, purchased from TA Instruments and were hermetically sealed containing 5 to 10 mg of sample. Polymers were subjected to three heating and cooling cycles at 10 °C/min. The glass transition temperature of each polymer was reported based on the onsets from the second heating curve.

#### 4.3.5 Thermogravimetric Analysis (TGA)

Thermogravimetric analysis was performed using a TA Instruments Discovery thermogravimetric analyzer in the Soft Matter Characterization Facility at the University of Chicago. Samples (∼5–10 mg) were heated from approximately 35 °C to 600 °C at a heating rate of 10 °C/min.

### 4.4 Electrolyte Preparation

The polymers were dissolved in 0.1 M TBAPF_6_ in DMF. Specifically, stock solutions (90 mM of PVBP, 84 mM of PEPP, and 67.5 mM of PMAP) were prepared with stirring under inert atmosphere at 80 °C until completely dissolved. Concentration was calculated according to the molar mass of the repeat unit. Lower concentration samples were obtained by serial dilution of the stock solution with electrolyte.

### 4.5 Electrochemical Characterization

A three-electrode beaker cell with a 3 mm (about 0.12 in) diameter glassy carbon electrode (GCE) working electrode, an Ag/AgNO_3_ (0.1 M AgNO_3_ solution) reference electrode, and a platinum wire counter electrode was used. All reported potentials are referenced to the Ag/Ag^+^ redox couple. Cyclic voltammetry and chronocoulometry were conducted in an argon atmosphere using a Gamry Interface-100 electrochemical workstation. Cyclic voltammetry was carried out after measuring the open circuit potential (ca. −0.6 V to −0.7 V) for scan rates 5, 10, 20, 30, 40, 50, 100, and 200 mV/s. The initial scan was initiated from OCP to −2.5 V, followed by the reverse scan to −1.5 V. All reported CVs correspond to the second cycle, scanned between −1.5 V and −2.5 V starting from −1.5 V. Chronocoulometry was performed by applying three potential steps: −1.5 V for 0.5 s, −2.5 V for 2 s, and finally −1.5 V for 2 s. *D_app_* values were calculated using the Anson equation.^52,53^ The kinetic rate constants *k*_ex_ and *k^0^* were obtained using the Dahms-Ruff equation^55,56^ and simplified Nicholson method, respectively. For the Dahms-Ruff equation, the concentration (C_0_*) was taken as the repeat unit concentration in the electrolyte, and the phthalimide site-to-site distance (δ) was taken as 6.75 nm (obtained computationally in our previous work^39^). In the Nicholson method, the dimensionless rate parameter, Ψ,^57^ was correlated with the peak separation at 5 mV/s. For PMAP and PVBP at 3c*, where irreversible redox behavior was observed, Ψ values were determined using the extended Lavagnini equation with an assumed electron transfer coefficient (α) of 0.5.^58^

### 4.6 Characterization of Viscosity

The viscosity measurements of PVBP and PMAP at varying concentrations were performed on a TA Instruments Rheometer HR-2 at Texas A&M University. Due to the very low viscosities of PMAP and PVBP in the electrolyte, a double-gap concentration configuration was used. The viscosity readings of PEPP were obtained in a cone and plate configuration on the same viscometer. The overlap concentration, c*, was taken as the intersection of two tangent lines of the low and high concentration regimes in a log viscosity vs concentration plot.^27^

### 4.7 Computational Experiments

#### 4.7.1 Molecular Dynamics Simulation:

To investigate polymer conformations in the solution state, we performed temperature-based replica exchange molecular dynamics (T-REMD) simulations over a temperature range of 250–350 K.^59,60^ Simulations were conducted on single polymer chains consisting of either 10 or 20 repeat units, each solvated in an explicit electrolyte solution containing molecular solvent and dissolved salt. All three polymer chemistries—PMAP, PVBP, and PEPP—were studied under identical conditions. Simulation box dimensions were chosen to accommodate the polymer in a fully extended conformation while ensuring that the minimum polymer–image separation exceeded the 1.1 nm nonbonded cutoff, thereby avoiding self-interactions across periodic boundaries. The resulting box sizes were approximately 4.5 × 4.5 × 4.5 nm^3^ for the 10-mer systems and 6.5 × 6.5 × 6.5 nm^3^ for the 20-mer systems.

To quantify concentration effects on polymer mobility and to reduce finite-size artifacts in diffusion estimates,^61^ additional 10-mer systems were prepared in a larger cubic box (10 nm side length) by varying the number of polymer chains while keeping the electrolyte composition fixed. Specifically, systems containing 1 chain (diluted) or 5 chains (semidiluted) were constructed, corresponding to concentrations of 16.6 mM (diluted) and 83.0 mM (semidiluted), respectively.

The force field for the polymers and electrolyte ions is taken from our previous work on phthalimide-based polymers.^39^ Polymer chains were described using the OPLS-AA/CM1A force field, with bonded parameters and CM1A-derived partial atomic charges assigned via LigParGen.^62,63^ Each polymer was terminated with methyl groups at both ends and polymer topologies were constructed using the Polyply toolkit.^64^ TBA^+^ and PF_6_^–^ ions were modeled using OPLS-AA-based parameters derived from the Canongia Lopes and Pádua force field.^65^ To improve the physical accuracy of these non-polarizable models, uniform charge scaling factor of 0.8 × q was applied to all ionic species, following the approach of Doherty *et al.* ^66^ A target electrolyte concentration of 0.1 M was achieved by inserting the appropriate number of ion pairs into each simulation box prior to solvation. The remaining volume was filled with DMF solvent, which was modeled using OPLS-AA parameters previously validated against experimental thermophysical properties of organic liquids, including density, dielectric constant, and enthalpy of vaporization, as reported by Caleman *et al.* ^67^

All simulations were performed using GROMACS 2022.4.^68^ After energy minimization, each system was equilibrated at its assigned temperature using a two-step protocol: 2 ns in the NVT ensemble with a velocity-rescaling thermostat (τ_T_ = 0.1 ps), followed by 5 ns in the NPT ensemble at 1 bar using a Berendsen barostat (τ_P_ = 1 ps).

Production simulations were carried out using T-REMD. Each 10-mer system was simulated with 24 replicas, while 20-mer systems used 36 replicas, with temperatures exponentially distributed between 250 and 350 K to ensure sufficient energy overlap and target an average acceptance rate of 20–30% between neighboring replicas. Exchange attempts were performed every 1 ps using the Metropolis criterion. Temperature and pressure during production were maintained using a Nosé–Hoover thermostat (τ_T_ = 1 ps) and a Parrinello–Rahman barostat (τ_P_ = 5 ps), respectively. Each replica was simulated for 150 ns in the smaller systems and 100 ns in the larger ones.

In addition, production simulations were performed for the concentration-series (diluted and semidiluted) 10-mer systems using conventional MD at 300 K. These runs employed the same thermostat and barostat settings (Nosé–Hoover, τ_T_ = 1 ps; Parrinello–Rahman, τ_P_ = 5 ps) and were simulated for 200 ns each

All simulations used a 1 fs integration time step, with bonds involving hydrogen atoms constrained via the LINear Constraint Solver (LINCS) algorithm. Long-range electrostatic interactions were calculated using the particle mesh Ewald (PME) method with a real-space cutoff of 1.1 nm. van der Waals interactions were truncated at the same distance, and analytical dispersion corrections were applied to both energy and pressure.

#### 4.7.2 Apparent Diffusion Coefficient

The apparent diffusion coefficient, *D*_*app*_, was calculated by accounting for both the physical motion of redox-active units and the contribution from charge hopping.^1,39,45,69^ The total diffusion coefficient was defined as the sum of these two mechanisms. The total diffusion coefficient was defined as the sum of these two mechanisms.

2 $$D_{app}=D_{phys}+D_{e}$$

Here, *D*_*phys*_ represents the translational diffusion of the redox-active monomer unit, obtained directly from the monomer-center-of-mass mean square displacement using Einstein-Kubo relation while *D*_*e*_ captures effective diffusion arising from electron hopping between redox centers. A detailed description of the calculate of *D*_*e*_ is provided in our previous work.

## Supplementary Material



## Data Availability

The data underlying this study are openly available in figshare at https://figshare.com/s/d0f3a8bbb87afbf4b16d. Molecular dynamics models for the polymers are available in the Polyply library (https://github.com/polyply).

## Notes

NMR spectra; GPC-SEC; TGA, DSC, time evaluation of *R*_g_; full collection of cyclic voltammetry including tests on blank electrolytes; kinetic parameters of three polymers; table of kinetic parameters of three polymers and table of kinetic parameters of redox polymers/small molecules; and DLS of three polymers (PDF)
