# Supplementary material for: Deciphering Concentration and Backbone Identity Effects on the Redox Properties of Phthalimide-Based Polymers
Source: Chem Mater. 2026 Sep 14;38(18):9530–41. doi: 10.1021/acs.chemmater.6c01965 (PMC13602850; doi:10.1021/acs.chemmater.6c01965)
Supplement: Supplementary file 1 [file cm6c01965_si_001.pdf]

Supplementary information

Deciphering Concentration and Backbone Identity Effects on the Redox Properties of Phthalimide-based Polymers

Khirabdhi Tannaya Mohanty,[1+] Sheila Keating,[2+] Kha Trinh,[3,4] Riccardo Alessandri,[5] Daniel P. Tabor,[6] Stuart Rowan,*[2, 3, 7] Juan de Pablo, *[3, 4, 7, 8, 9] and Jodie L. Lutkenhaus*[1, 10]

[1] Artie McFerrin Department of Chemical Engineering, Texas A&M University, TX 77843, United States of America

[2] Department of Chemistry, University of Chicago, Chicago, IL 60637, United States of America

[3] Pritzker School of Molecular Engineering, University of Chicago, IL, United States of America

[4] Department of Chemical and Biological Engineering, Tandon School of Engineering, New York University, Brooklyn, New York 11201, United States

[5] Department of Chemical Engineering, KU Leuven, Celestijnenlaan 200F, 3001 Leuven, Belgium

[6] Department of Chemistry, Texas A&M University, TX 77840, United States of America

[7] Department of Computer Science, Courant Institute of Mathematical Sciences, New York University, New York, NY 10012, USA

[8] Department of Physics, New York University, New York, New York 10012, United States

[9] Materials Science Division, Argonne National Laboratory, Lemont, Illinois 60439, United States

[10] Department of Materials Science and Engineering, Texas A&M University, TX 77840, United States of America

* Corresponding author

Email: jodie.lutkenhaus@tamu.edu

stuartrowan@uchicago.edu

j.de.pablo@nyu.edu

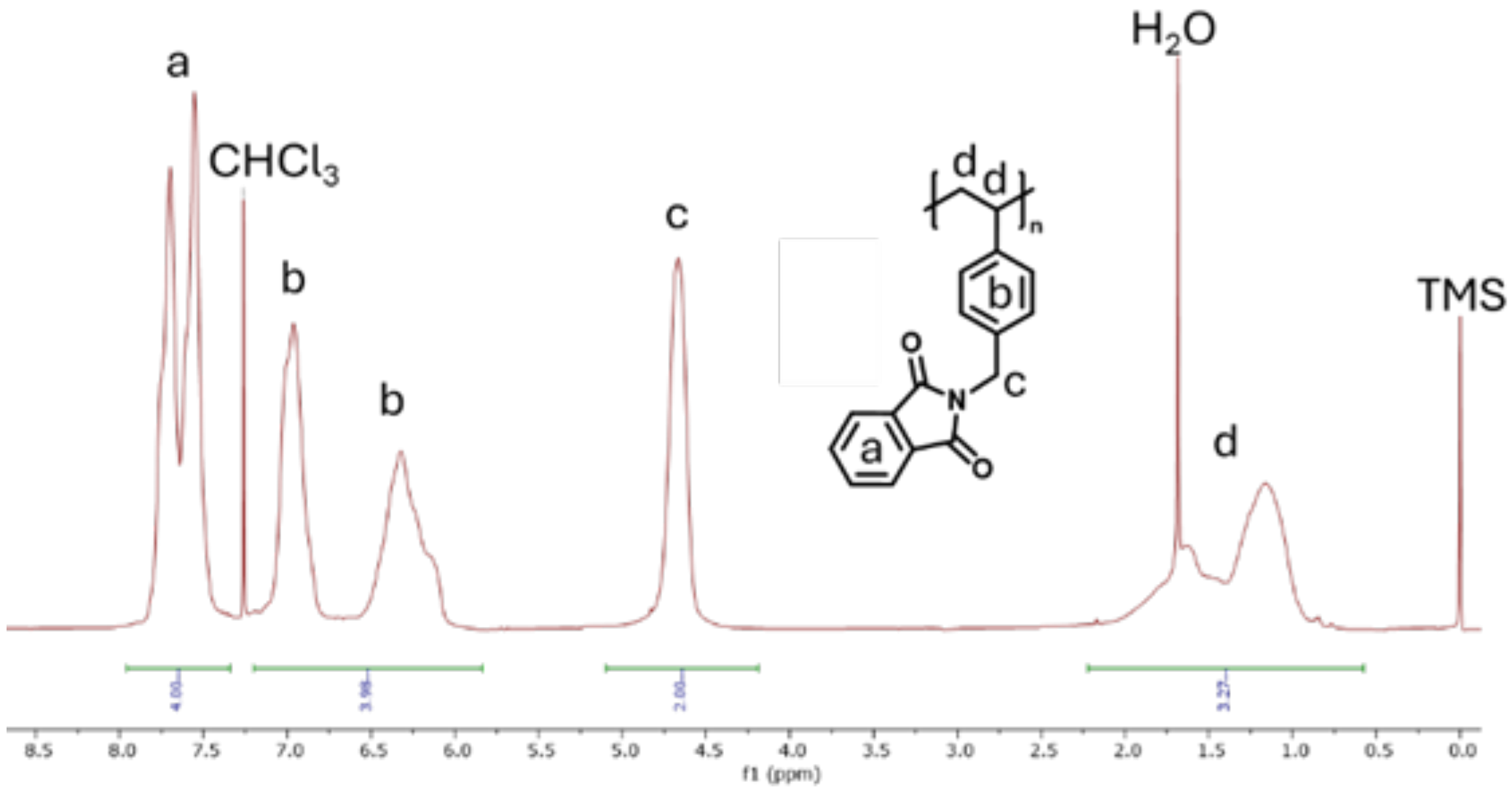

Figure S1. ^{1}H NMR spectrum of PVBP in $CDCl_3$.

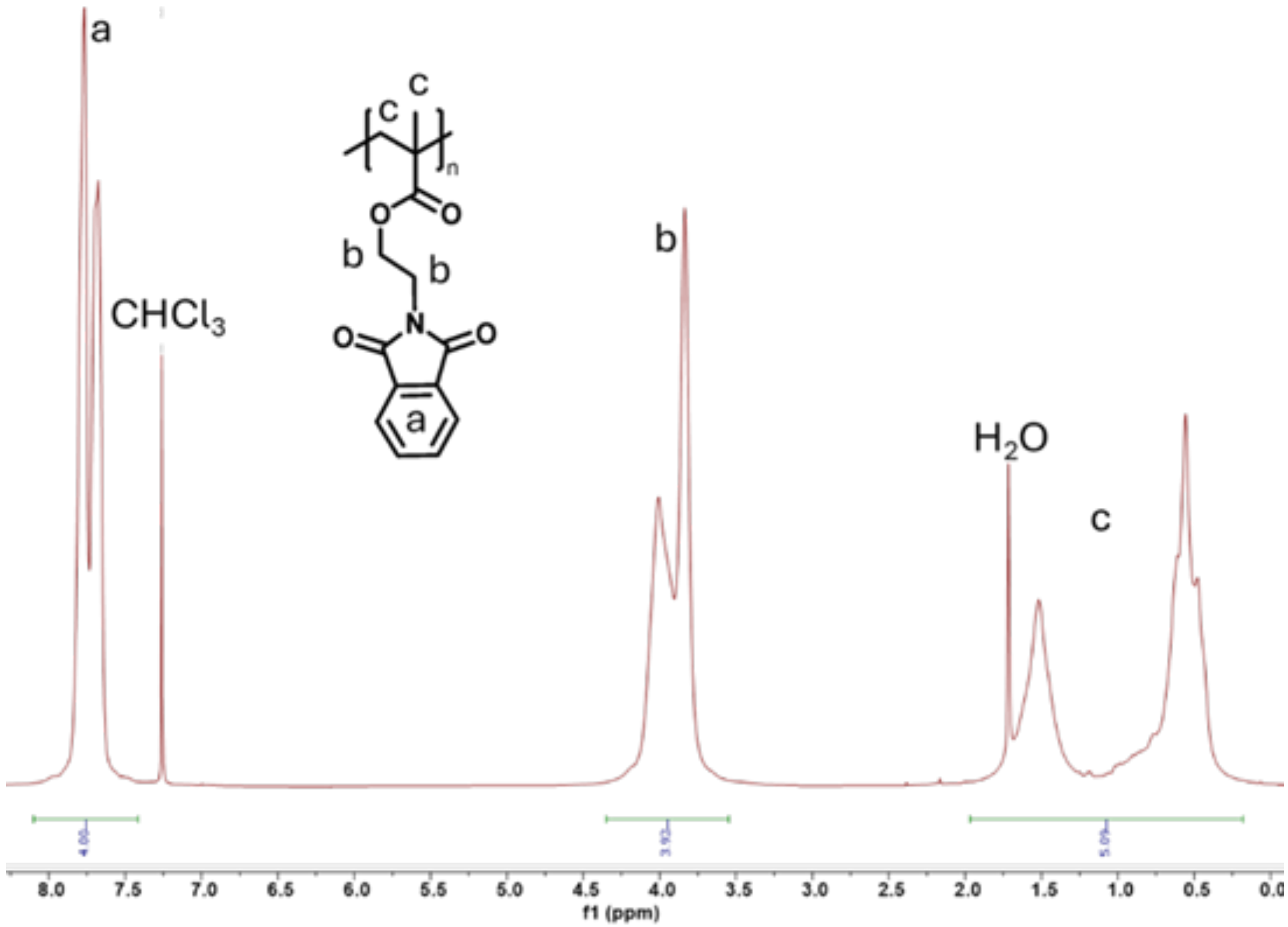

Figure S2. ^{1}H NMR spectrum of PMAP in $CDCl_3$.

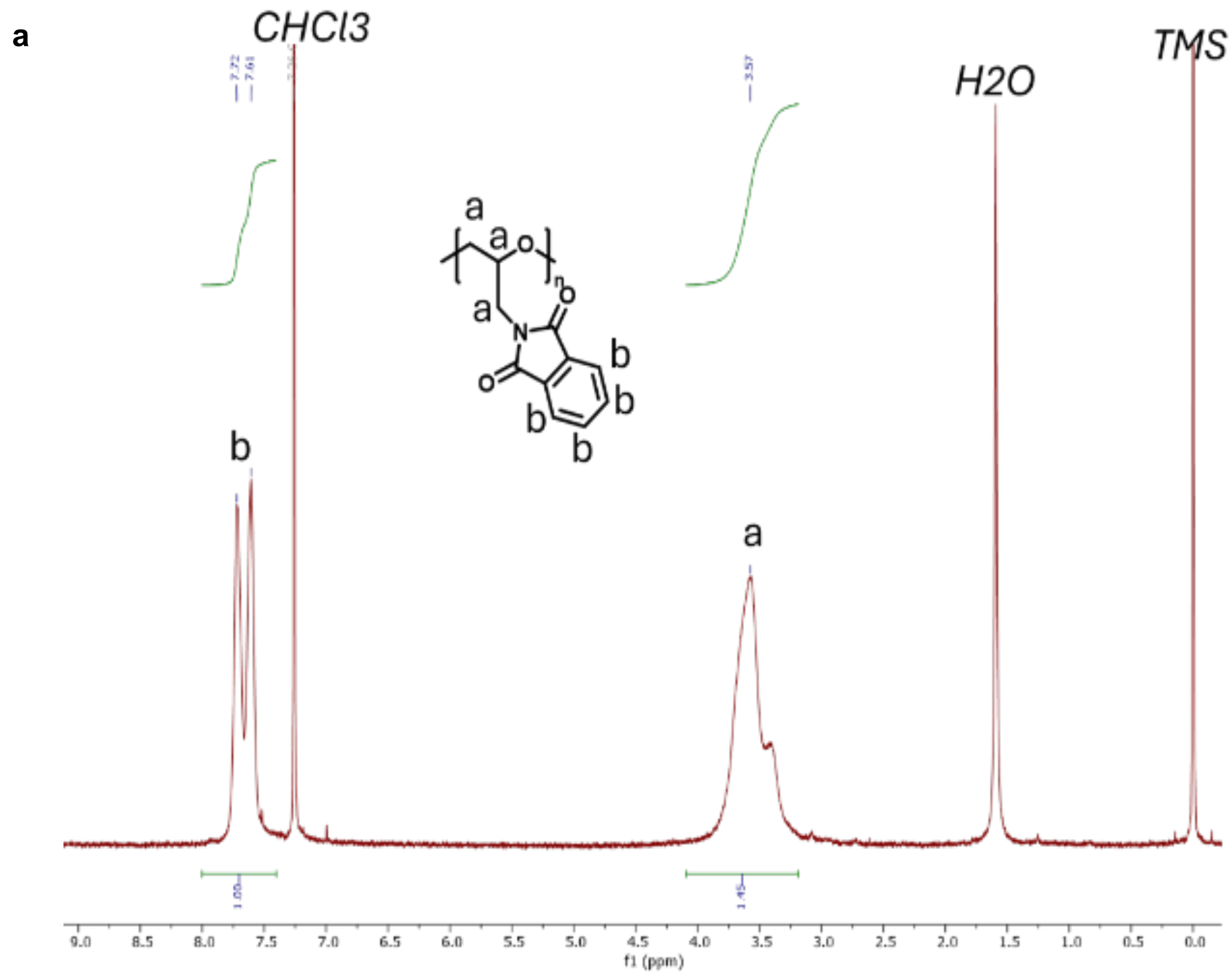

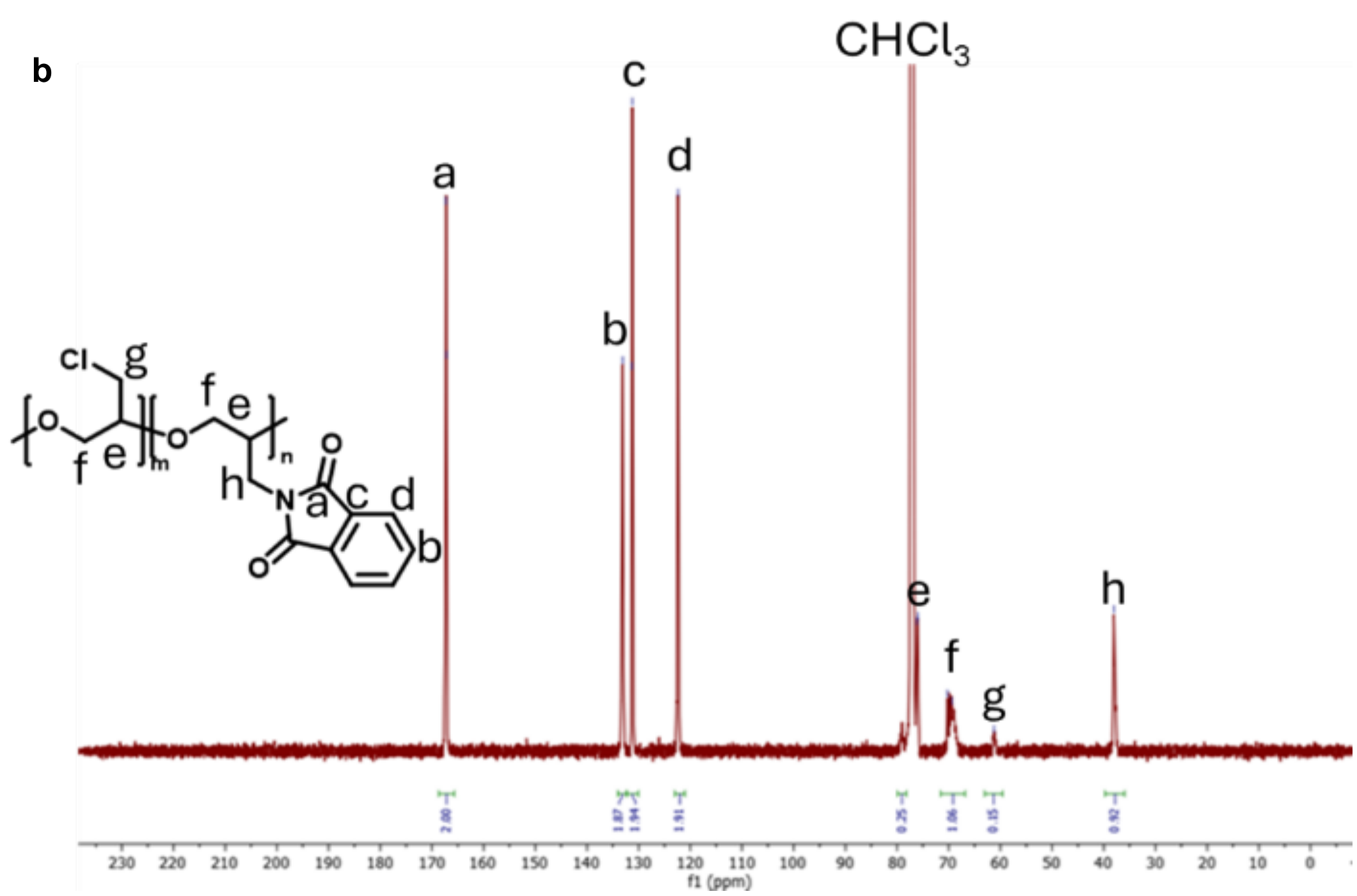

Figure S3. (**a**) ^{1}H NMR and (**b**) ^{13}C quantitative NMR spectra of PEPP in $CDCl_3$.

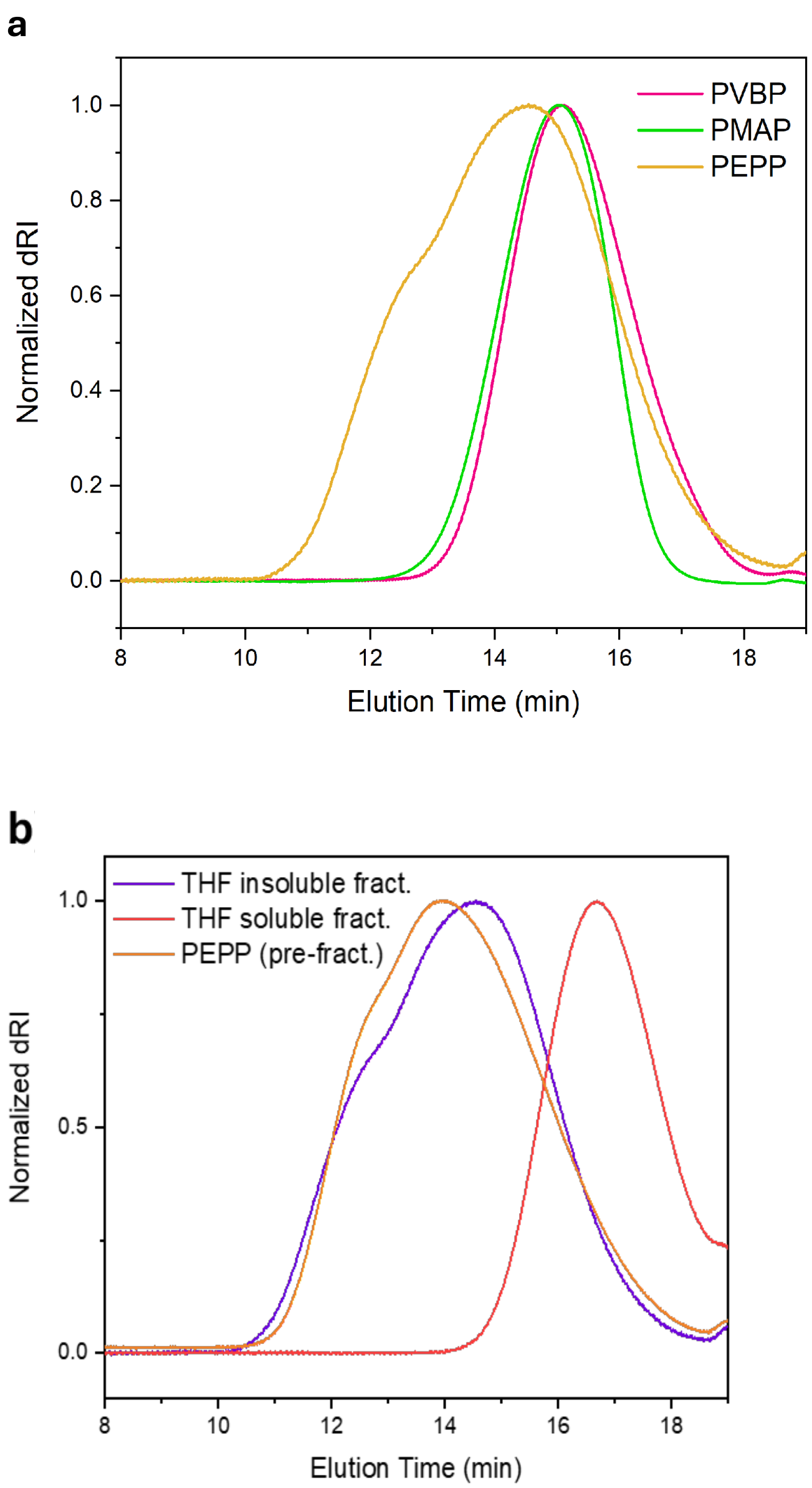

Figure S4. GPC-SEC profile of (a) the polyphthalimides and (b) PEPP fractionation by THF solubility, where the THF insoluble fraction is denoted as "PEPP" in the text.

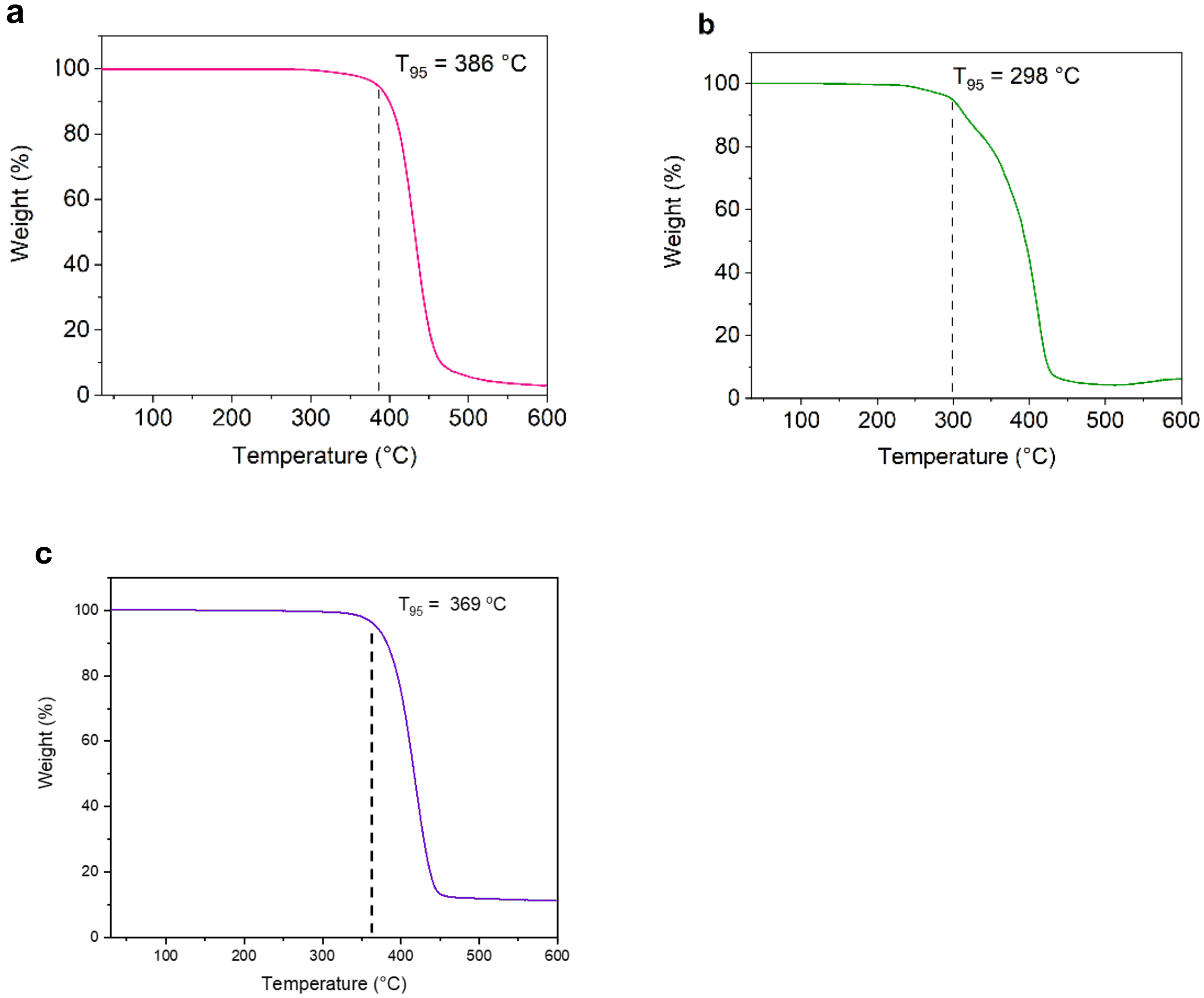

Figure S5. Thermogravimetric analysis (TGA) of (a) PVBP (b) PMAP (c) PEPP.

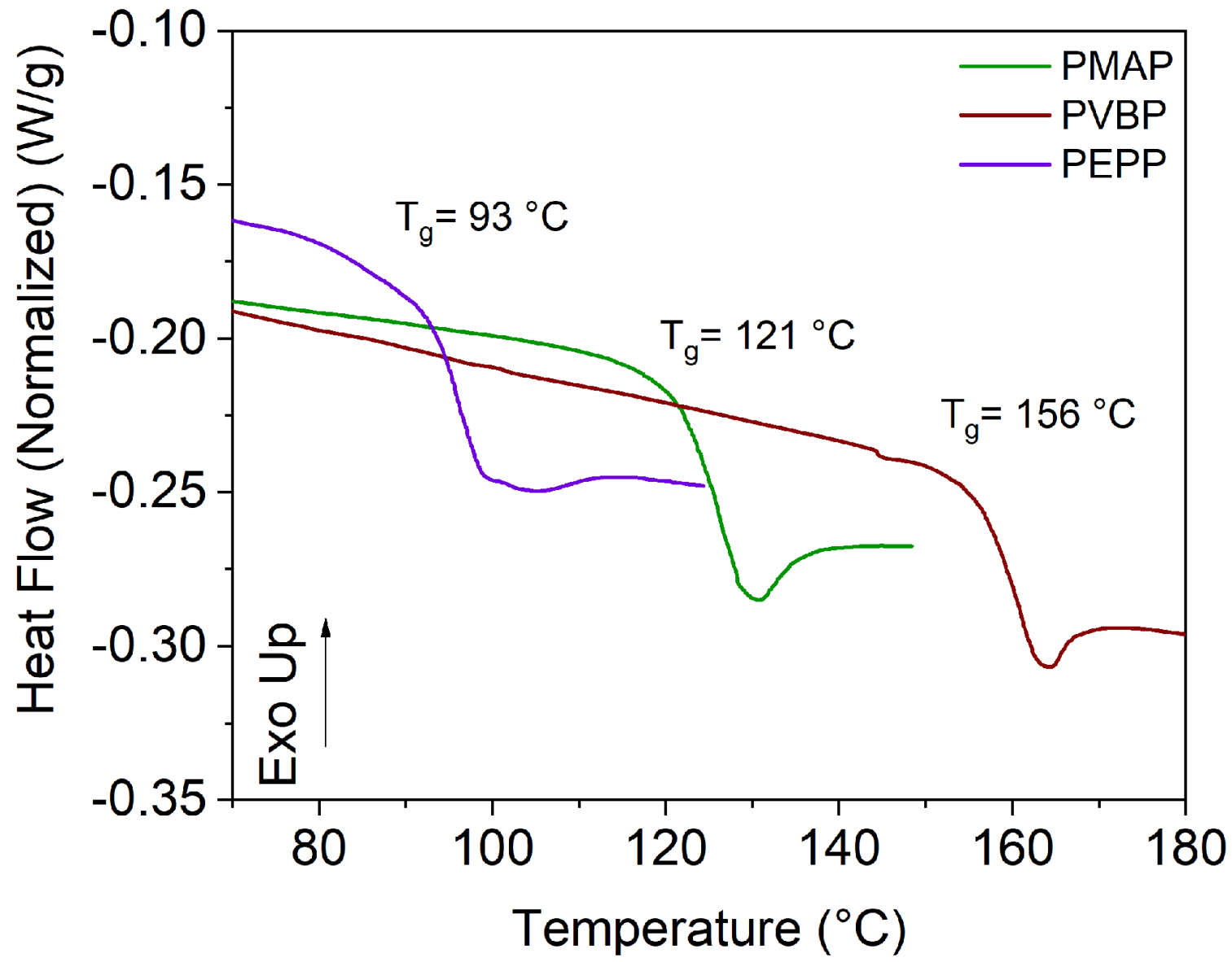

Figure S6. Differential scanning calorimetry (DSC) of PMAP, PVBP, and PEPP, showing the 2nd heating scan at 10 °C/min.

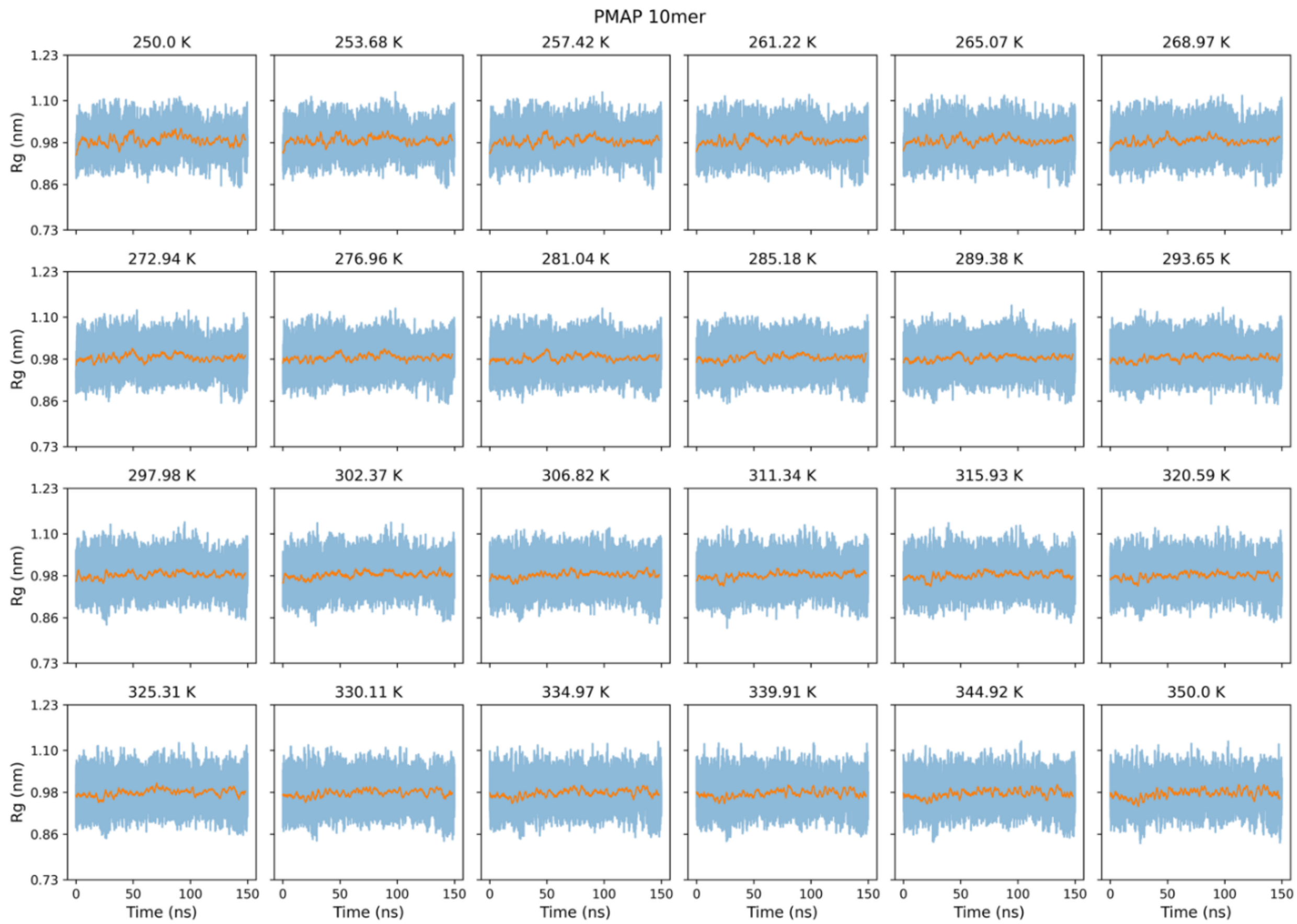

Figure S7. Time evolution of radius of gyration (R_g) for PMAP 10mer across all temperatures in replica exchange molecular dynamics simulations. The blue line shows the instantaneous R_g values at each time step, while the overlaid orange line represents the running average.

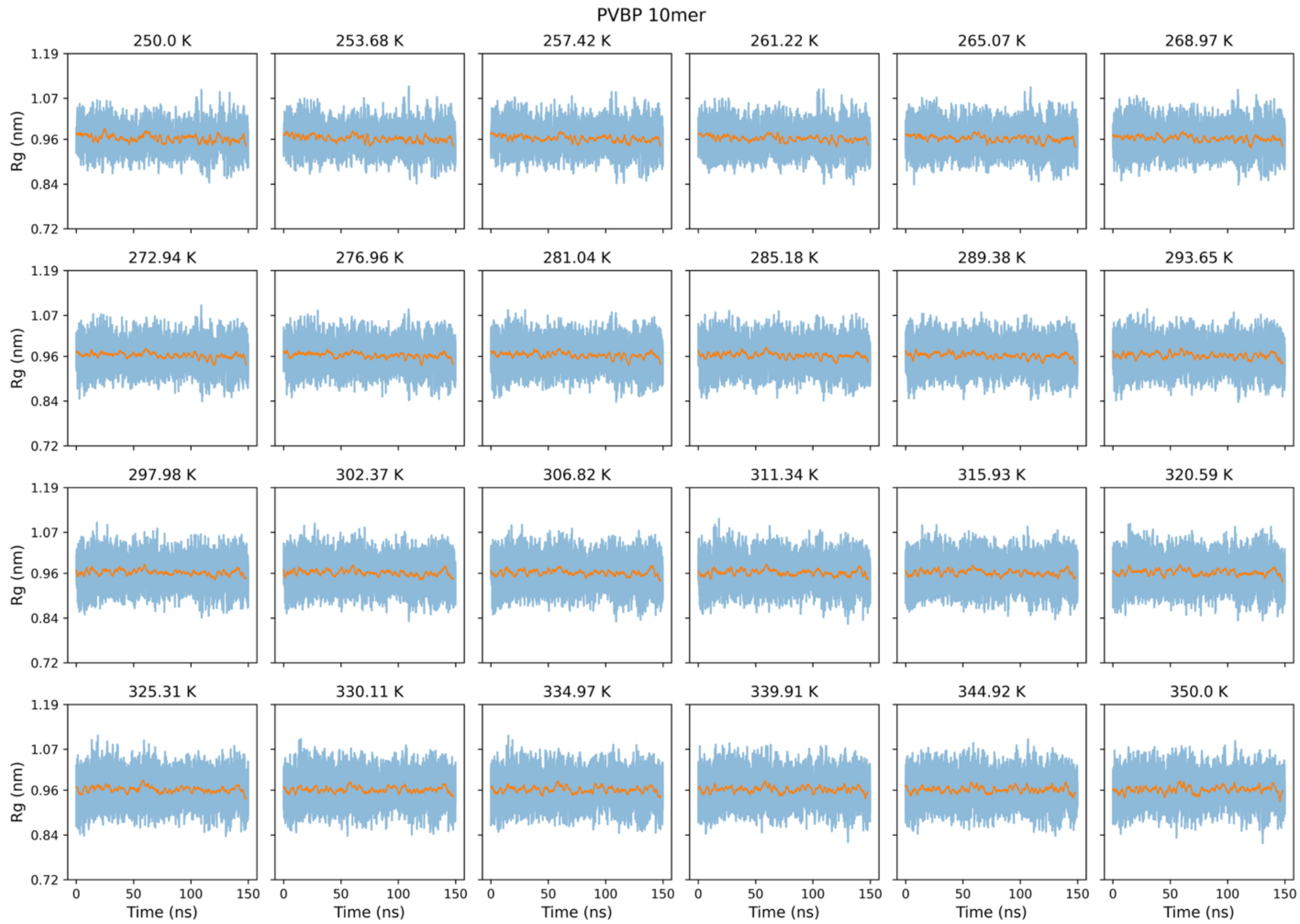

Figure S8. Time evolution of radius of gyration (R_g) for PVBP 10mer across all temperatures in replica exchange molecular dynamics simulations. The blue line shows the instantaneous R_g values at each time step, while the overlaid orange line represents the running average.

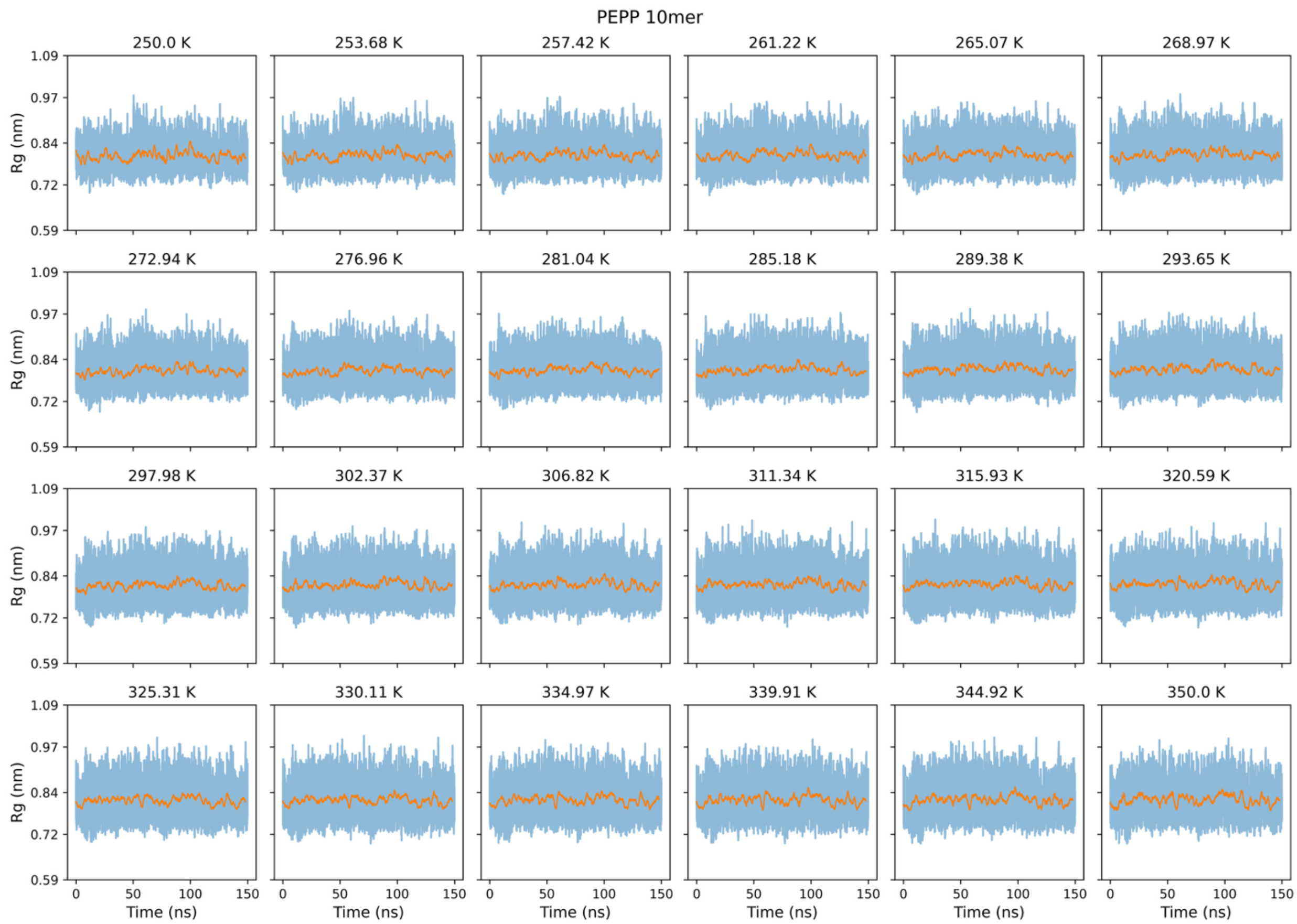

Figure S9. Time evolution of radius of gyration (R_g) for PEPP 10mer across all temperatures in replica exchange molecular dynamics simulations. The blue line shows the instantaneous R_g values at each time step, while the overlaid orange line represents the running average.

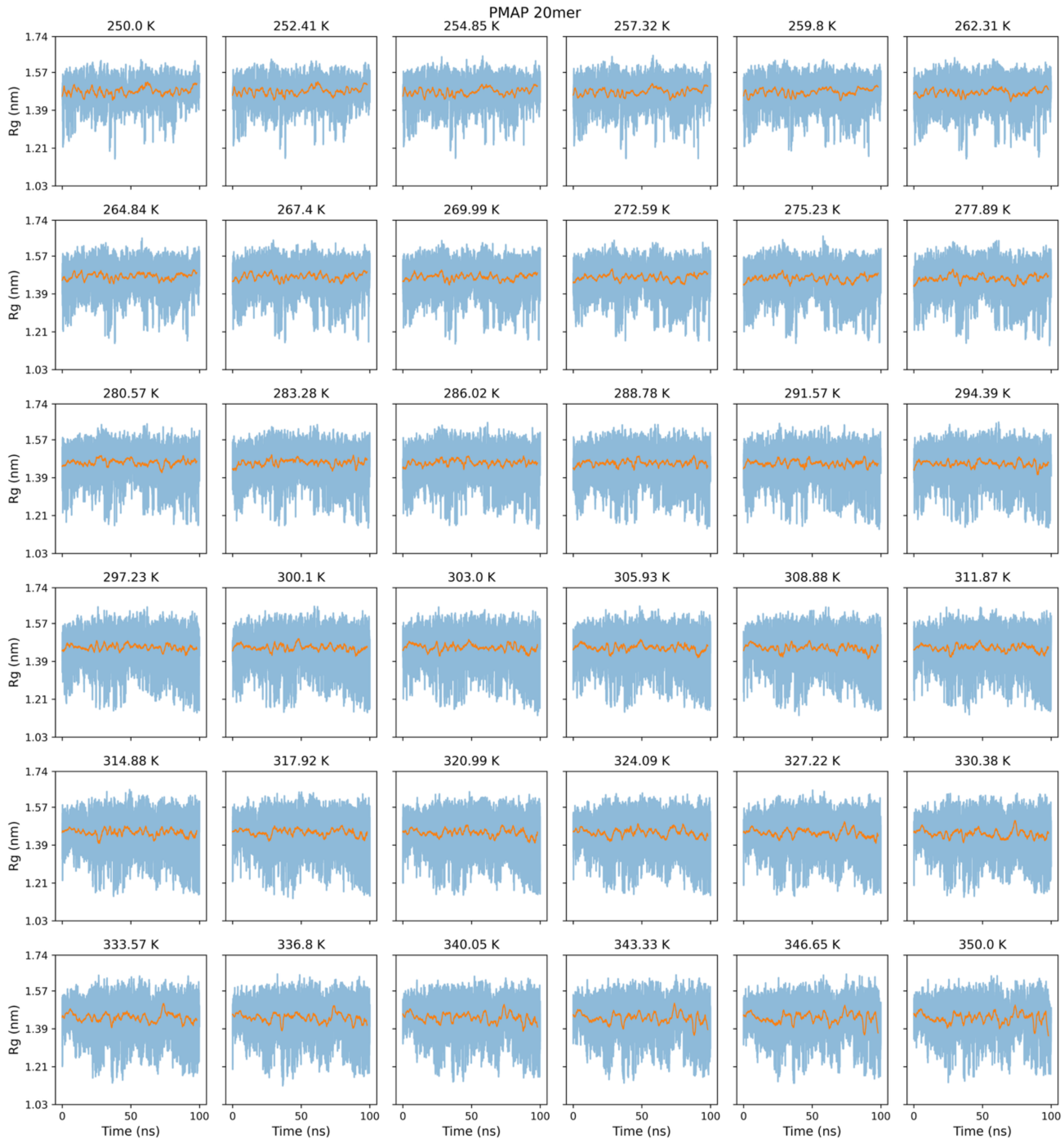

Figure S10. Time evolution of radius of gyration (R_g) for PMAP 20mer across all temperatures in replica exchange molecular dynamics simulations. The blue line shows the instantaneous R_g values at each time step, while the overlaid orange line represents the running average.

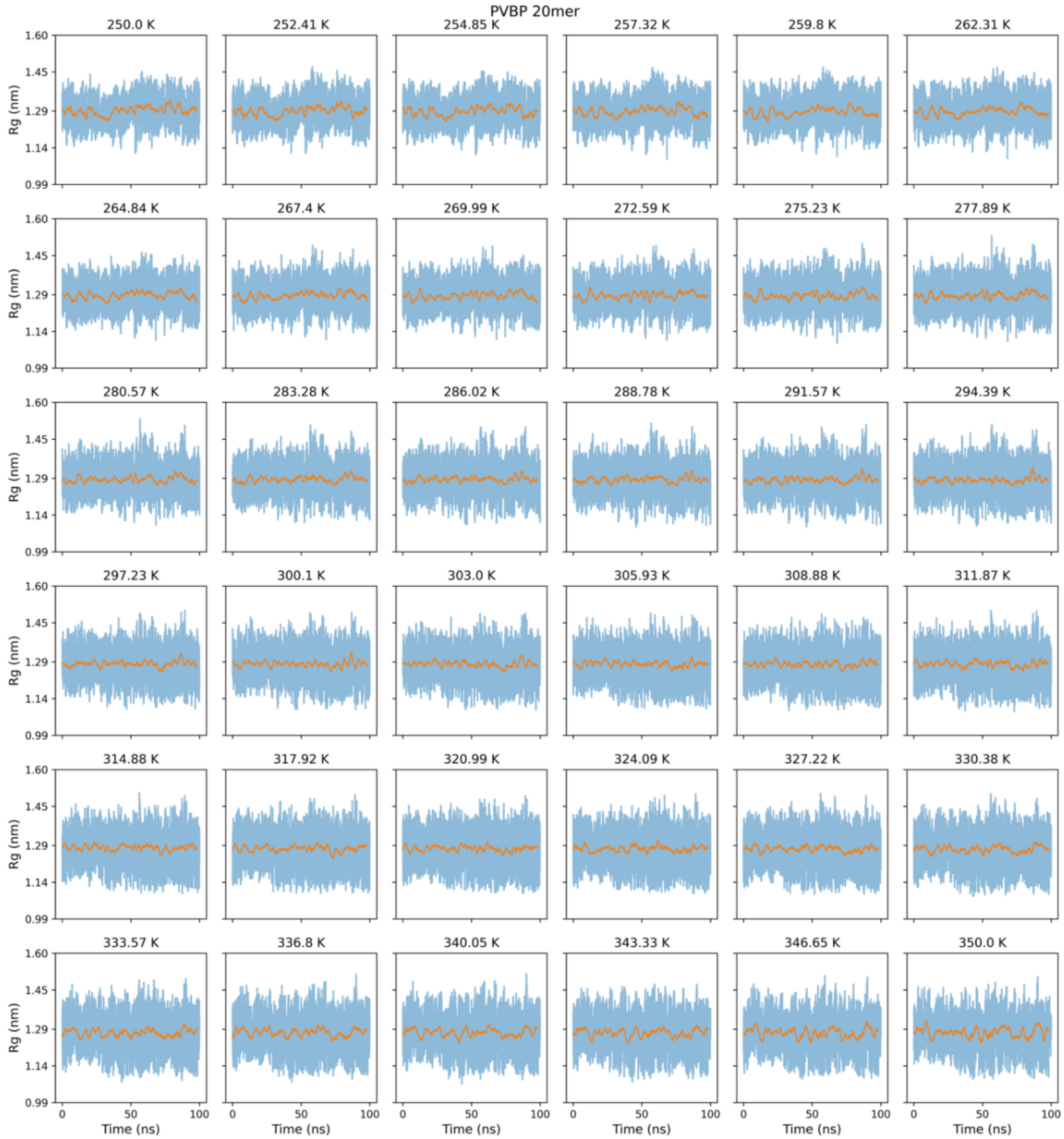

Figure S11. Time evolution of radius of gyration (R_g) for PVBP 20mer across all temperatures in replica exchange molecular dynamics simulations. The blue line shows the instantaneous R_g values at each time step, while the overlaid orange line represents the running average.

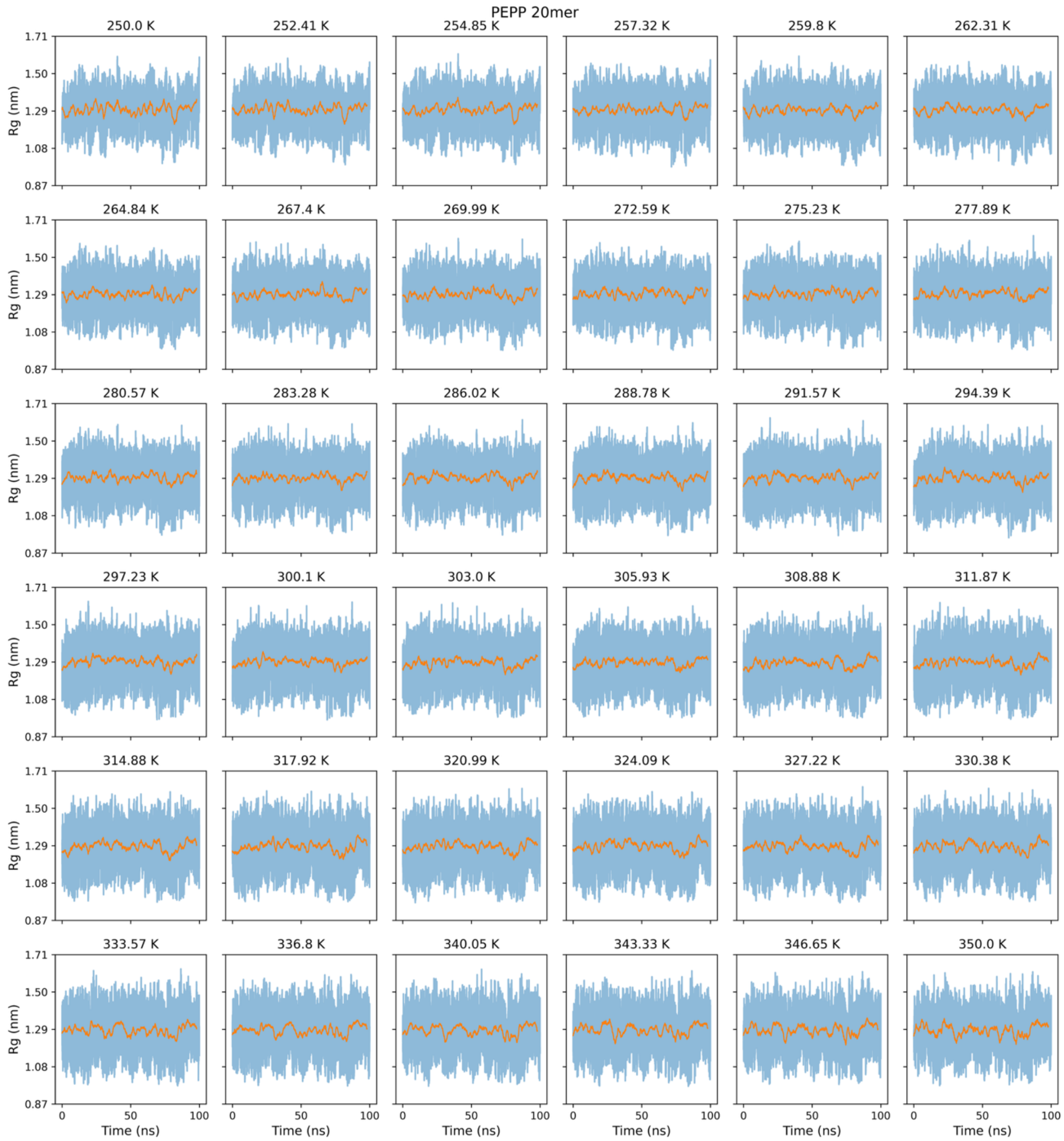

Figure S12. Time evolution of radius of gyration (R_g) for PEPP 20mer across all temperatures in replica exchange molecular dynamics simulations. The blue line shows the instantaneous R_g values at each time step, while the overlaid orange line represents the running average.

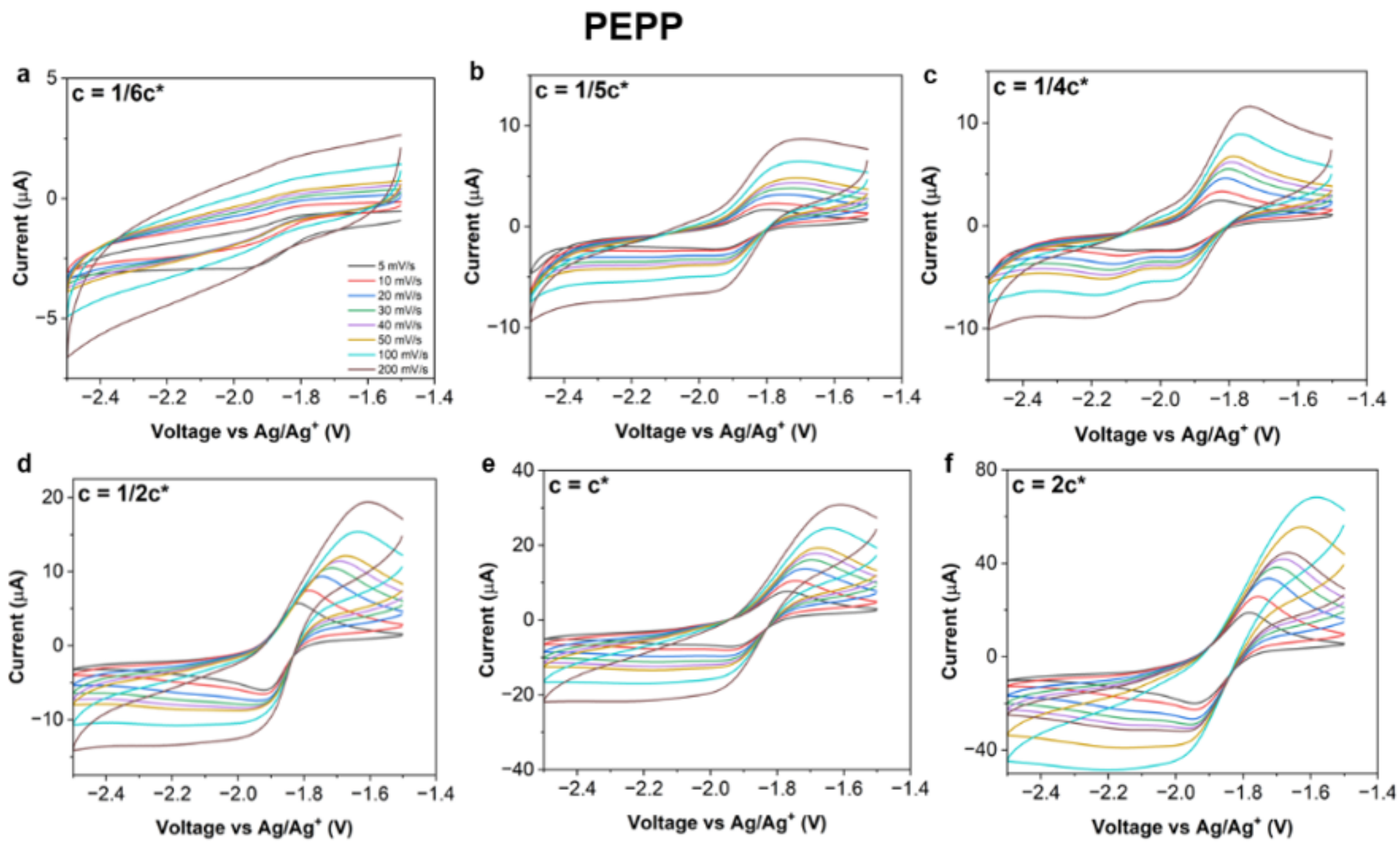

Figure S13. Cyclic voltammograms of PEPP in 0.1 M $TBAPF_6$ in DMF at 1/6c*, 1/5c*, 1/4c*, 1/2c*, c*, and 2c* obtained in a three-electrode beaker cell configuration (WE: GCE, RE: $Ag/AgNO_3$ and CE: pt wire) at different scan rates. The legend applies to all panels.

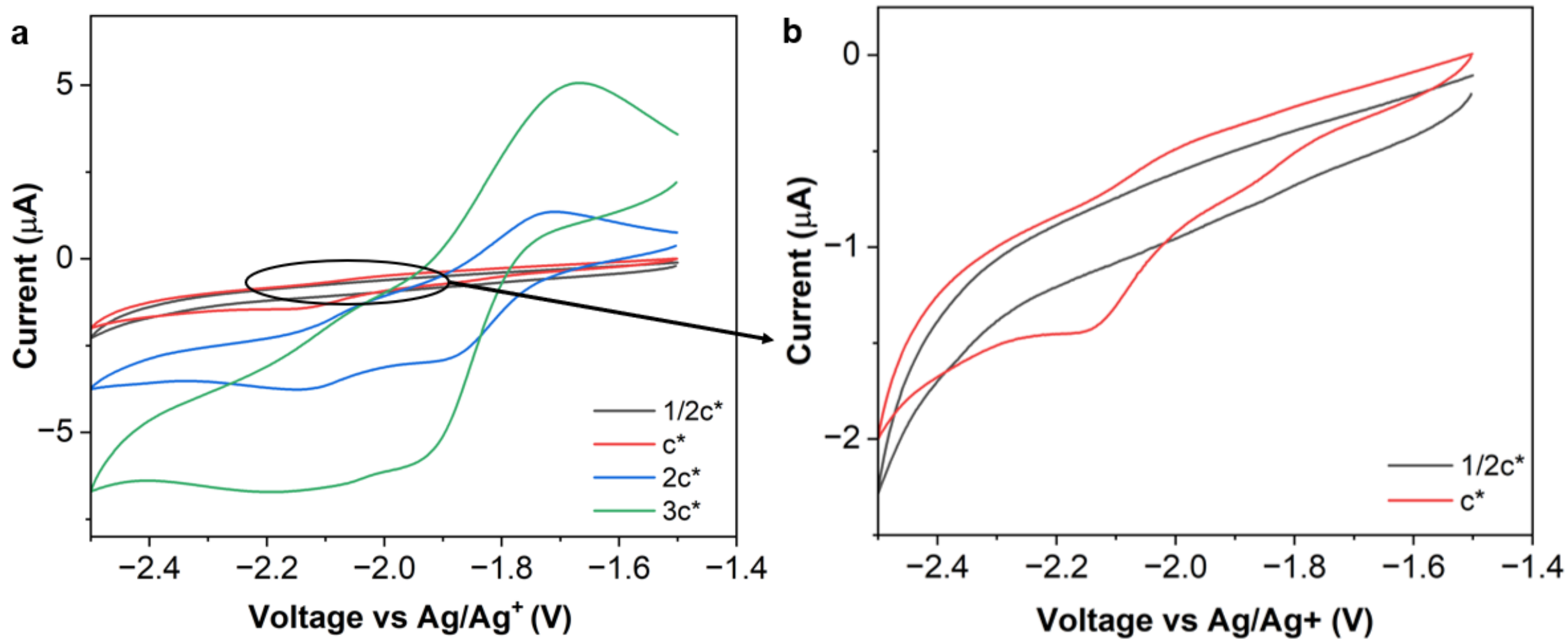

Figure S14. Cyclic voltammogram of a glassy carbon electrode in "blank" electrolyte, 0.1 M $TBAPF_6$ in DMF. Beforehand, the electrodes had been used in electrochemical tests of PEPP solutions (1/2c*, c*, 2c* and 3c*). Any electrochemical activity, therefore, is ascribed to adsorbed polymer from the prior experiment. A three-electrode beaker cell configuration (WE: GCE, RE: $Ag/AgNO_3$ and CE: pt wire) at 5 mV/s was used.

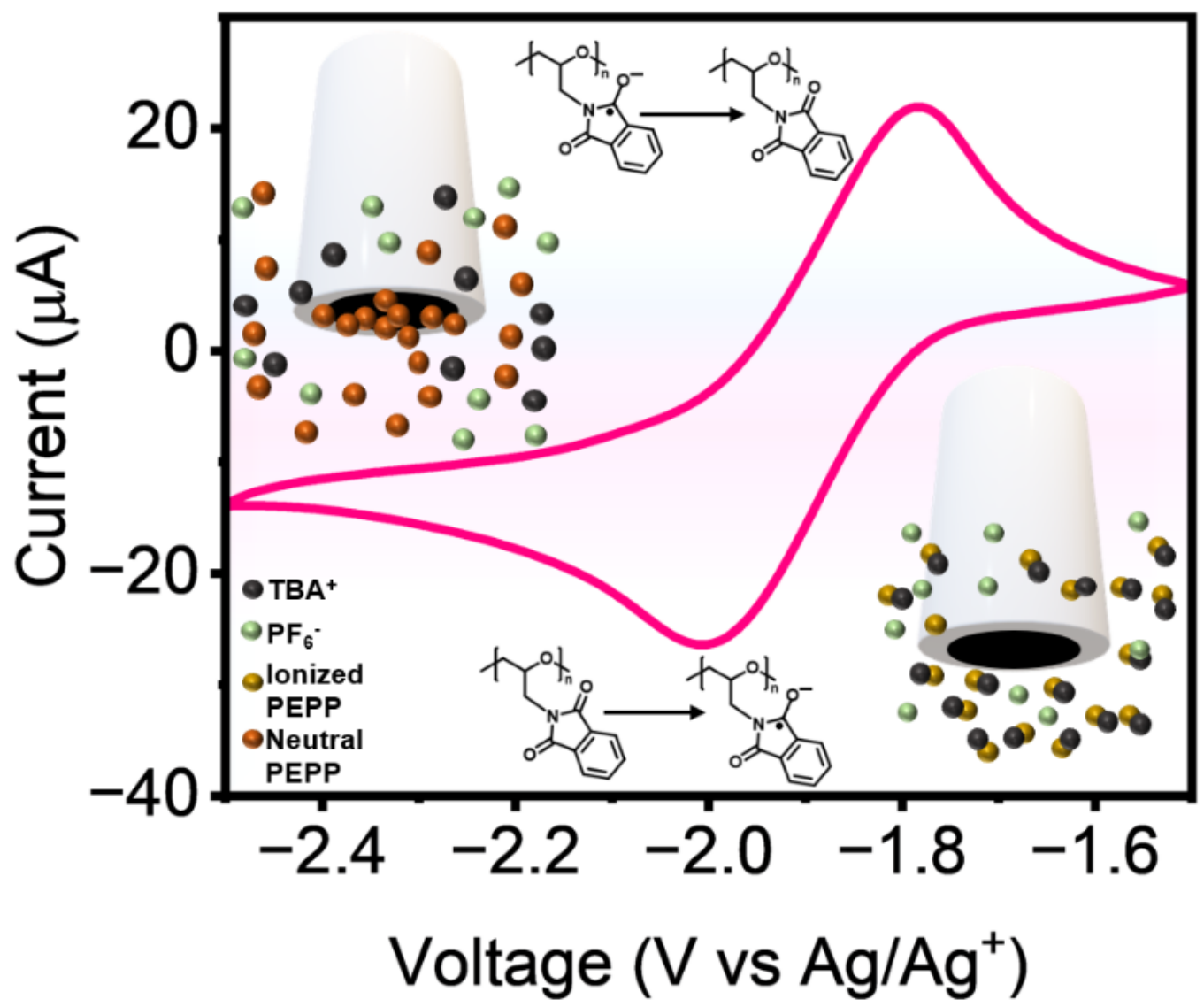

Figure S15. Schematic of PEPP adsorbing and stripping on an electrode surface during CV. The polymer solution was reduced during the reverse scan from –1.5 V to –2.5 V, and oxidized back to the neutral form upon application during the forward scan from –2.5 V to –1.5 V. A three-electrode beaker cell configuration (WE: GCE, RE: Ag/$AgNO_3$ and CE: pt wire) at 5 mV/s was used.

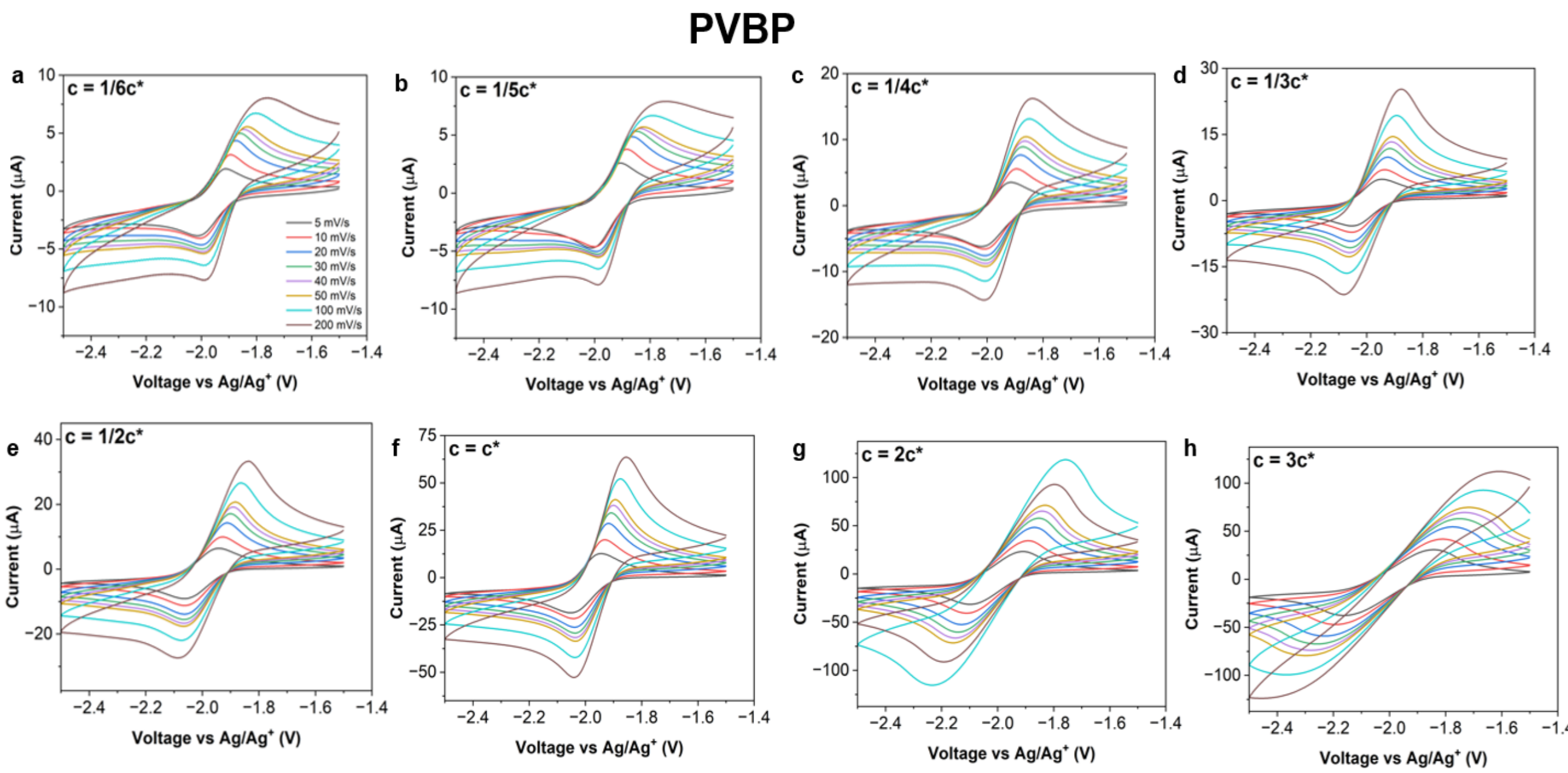

Figure S16. Cyclic voltammograms of PVBP in 0.1 M $TBAPF_6$ in DMF at 1/6c*, 1/5c*, 1/4c* and 1/3c*, 1/2c*, c*, 2c* and 3c* obtained in a three-electrode beaker cell configuration (WE: GCE, RE: $Ag/AgNO_3$ and CE: pt wire) at different scan rates. The legend applies to all panels.

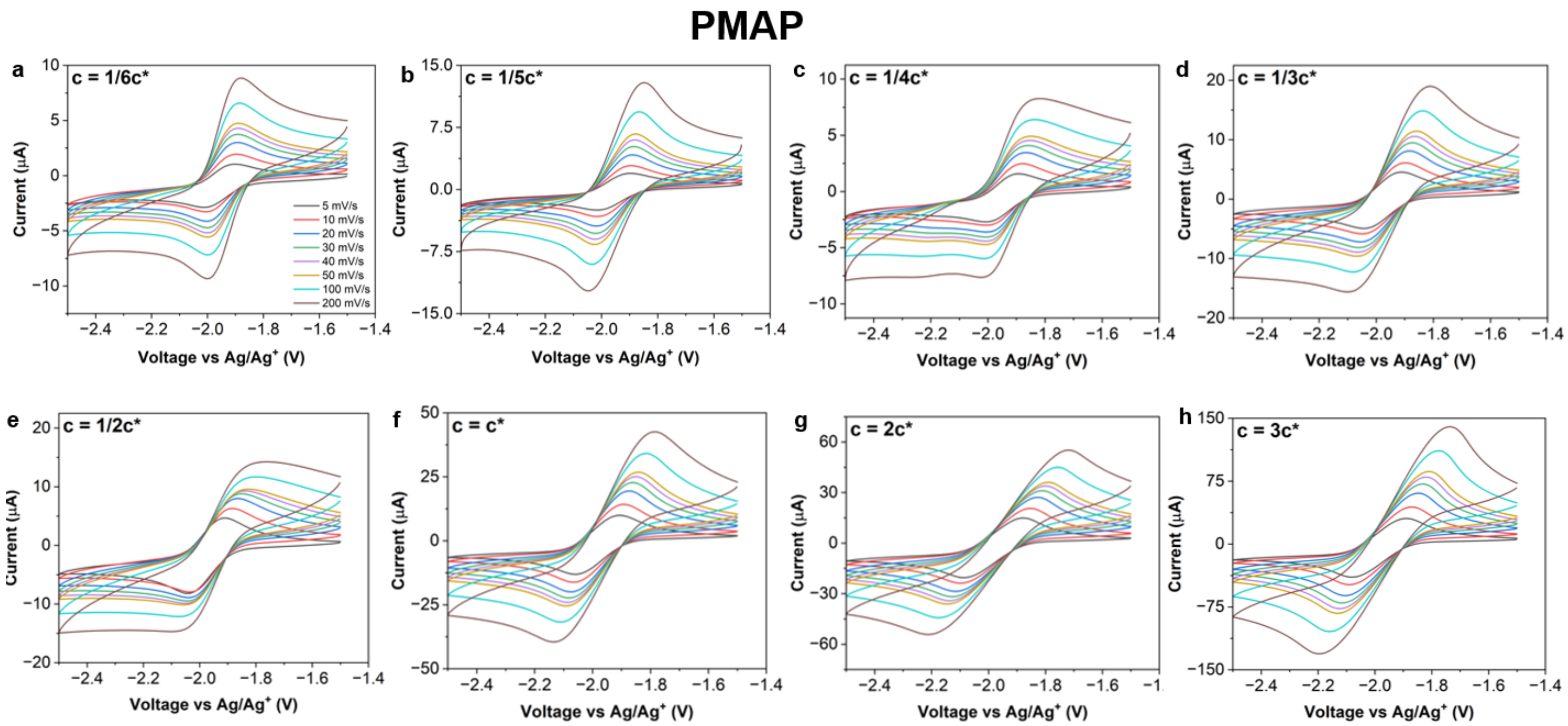

Figure S17. Cyclic voltammograms of PMAP in 0.1 M $TBAPF_6$ in DMF at 1/6c*, 1/5c*, 1/4c* and 1/3c*, 1/2c*, c*, 2c* and 3c* obtained in a three-electrode beaker cell configuration (WE: GCE, RE: $Ag/AgNO_3$ and CE: pt wire) at different scan rates. The legend applies to all panels.

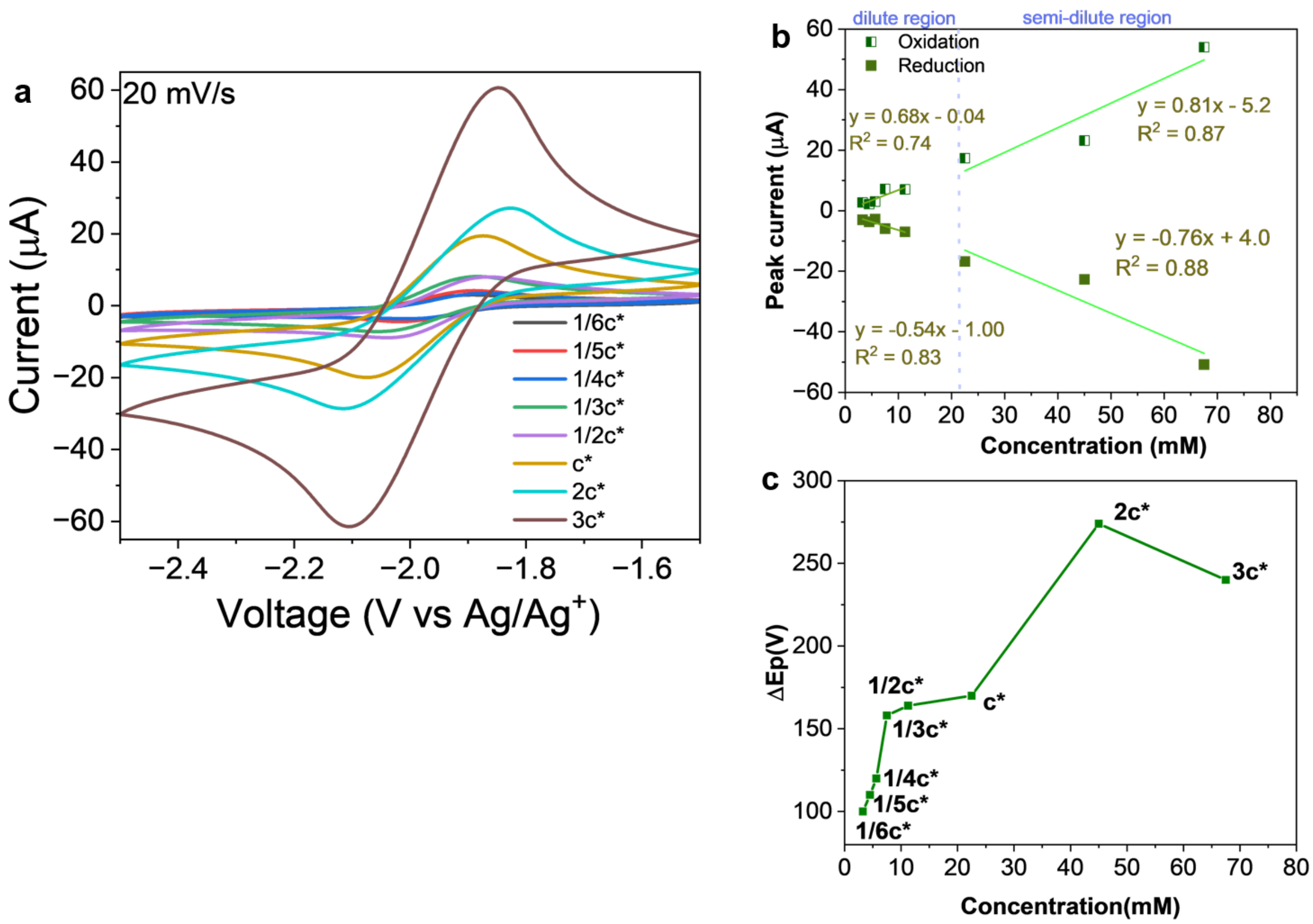

Figure S18. Cyclic voltammetry analysis at different concentrations of PMAP in 0.1 M $TBAPF_6$. a) Cyclic voltammograms at 20mV/s in a three-electrode beaker cell configuration (WE: GCE, RE: $Ag/AgNO_3$ and CE: Pt wire). b) Peak current and c) peak separation at different PMAP.concentrations.

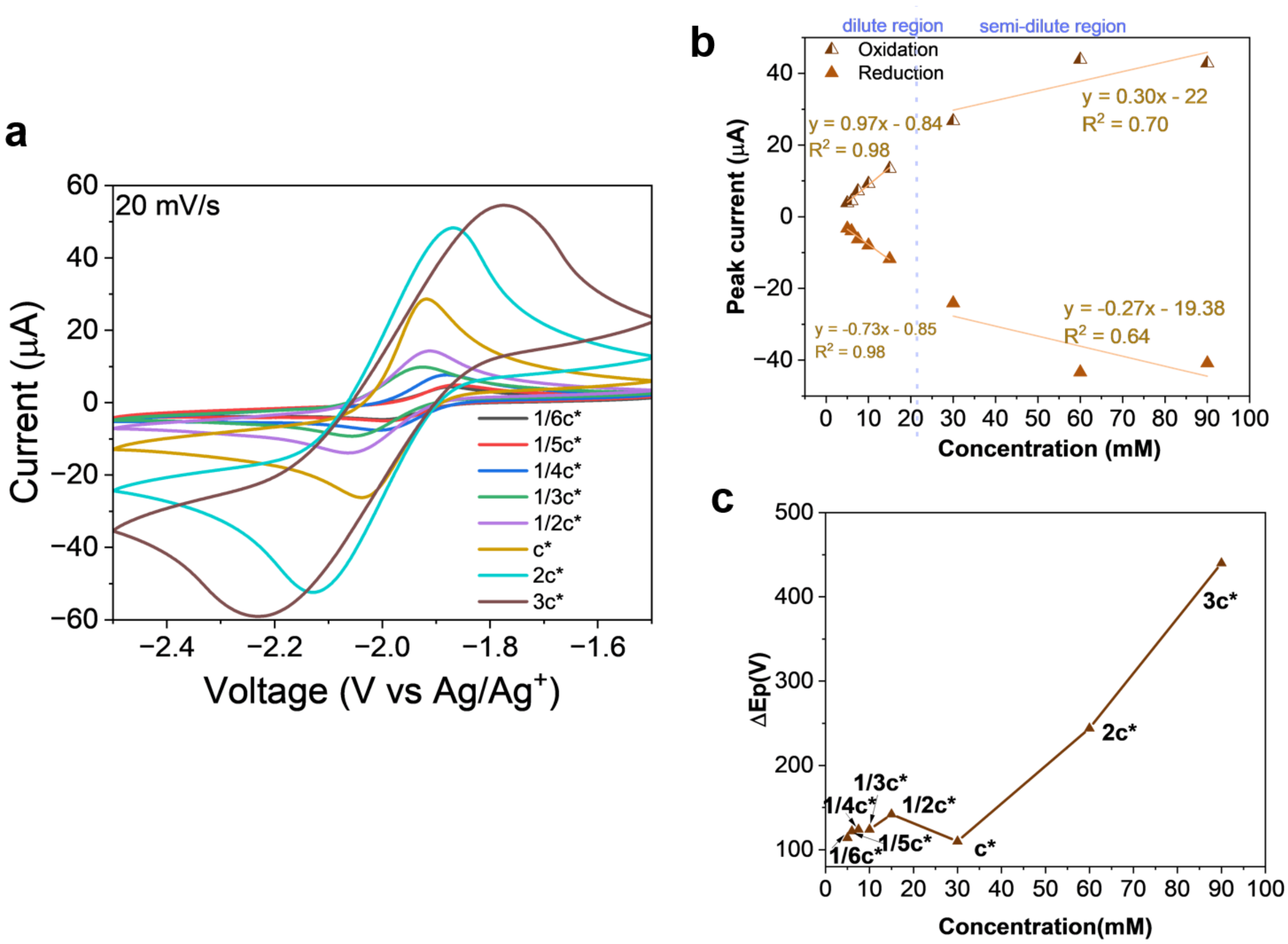

Figure S19. Cyclic voltammetry analysis at different concentrations of PVBP in 0.1 M $TBAPF_6$. a) Cyclic voltammograms at 20mV/s in a three-electrode beaker cell configuration (WE: GCE, RE: $Ag/AgNO_3$ and CE: Pt wire). b) Peak current and c) peak separation at different PMAP concentrations.

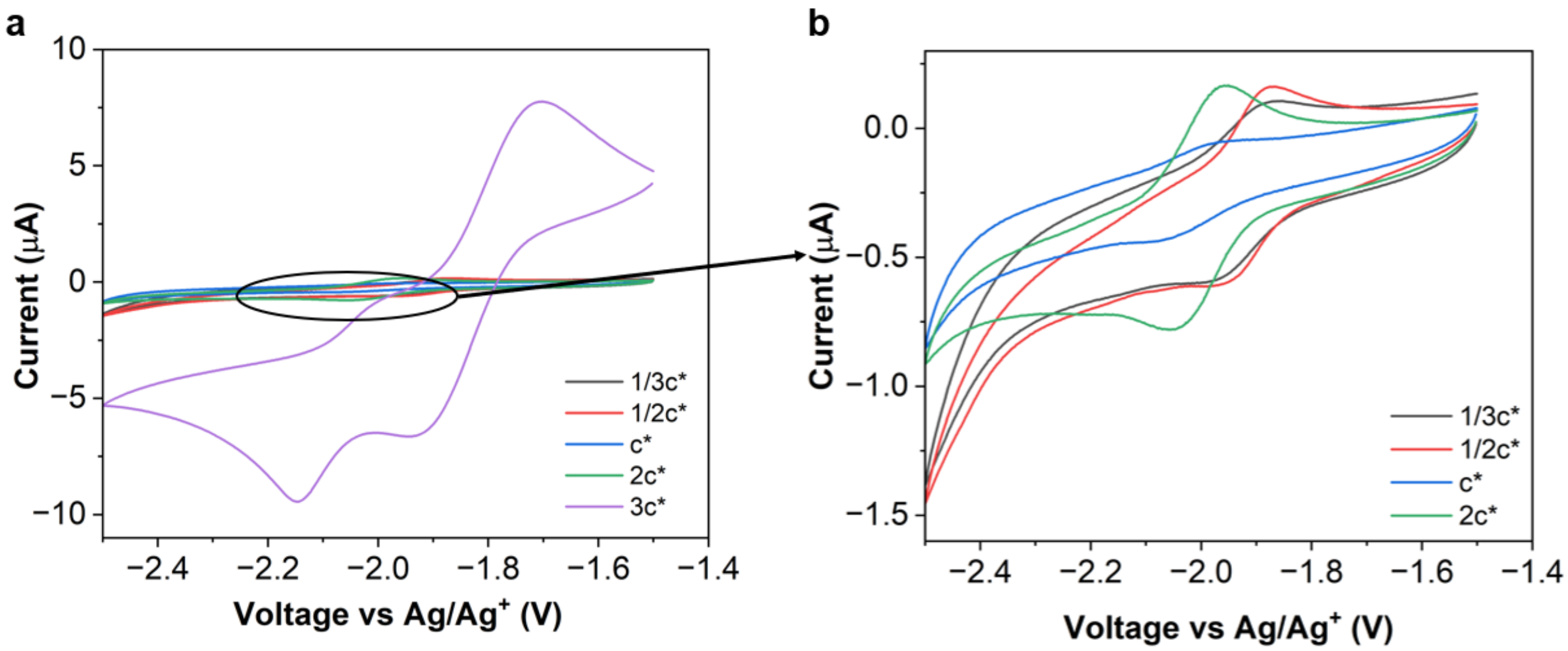

Figure S20. Cyclic voltammogram of a glassy carbon electrode in "blank" electrolyte, 0.1 M $TBAPF_6$ in DMF. Beforehand, the electrodes had been used in electrochemical tests of PVBP solutions (1/3c*, 1/2c*, c*, 2c* and 3c*). Any electrochemical activity, therefore, is ascribed to adsorbed polymer from the prior experiment. Minor adsorption was present 1/3c*, but major adsorption was evident at 3c*. A three-electrode beaker cell configuration (WE: GCE, RE: $Ag/AgNO_3$ and CE: pt wire) at 5 mV/s was used.

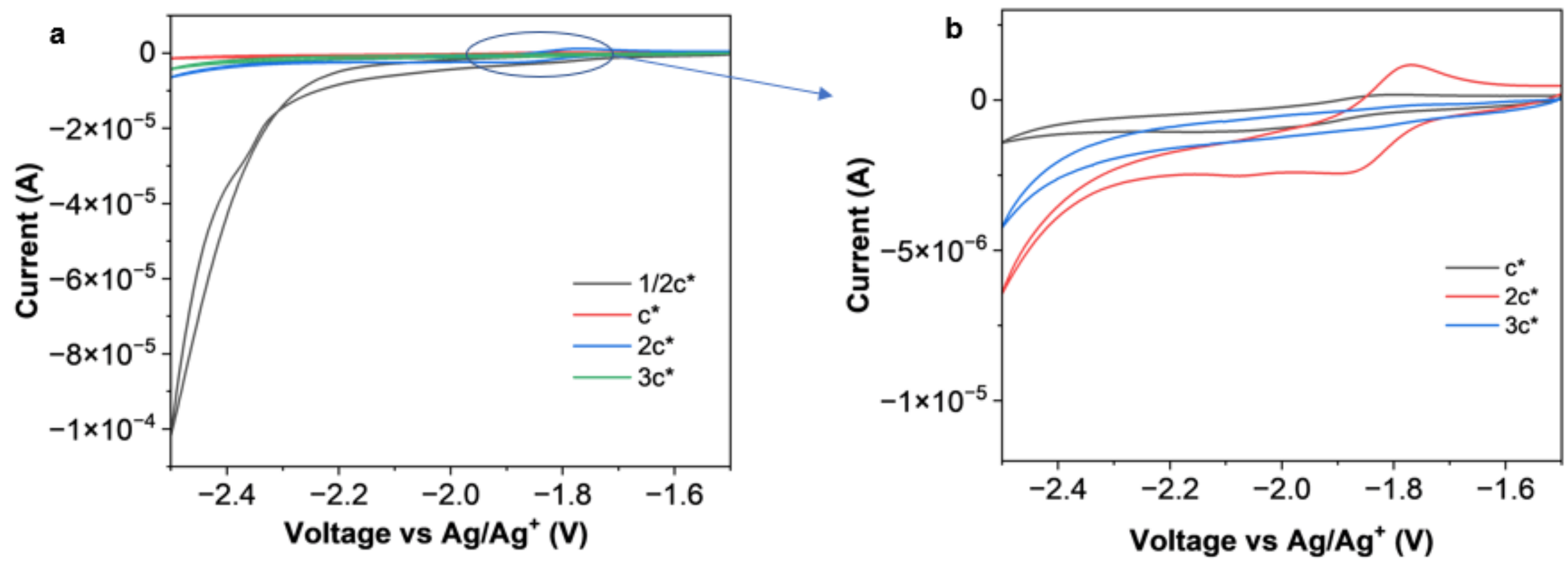

Figure S21. Cyclic voltammogram of a glassy carbon electrode in “blank” electrolyte, 0.1 M $TBAPF_6$ in DMF. Beforehand, the electrodes had been used in electrochemical tests of PMAP solutions (1/2c*, c*, 2c* and 3c*). Any electrochemical activity, therefore, is ascribed to adsorbed polymer from the prior experiment. Minor adsorption was present c*, but major adsorption was evident at 2c* and 3c*. A three-electrode beaker cell configuration (WE: GCE, RE: $Ag/AgNO_3$ and CE: pt wire) at 5 mV/s was used.

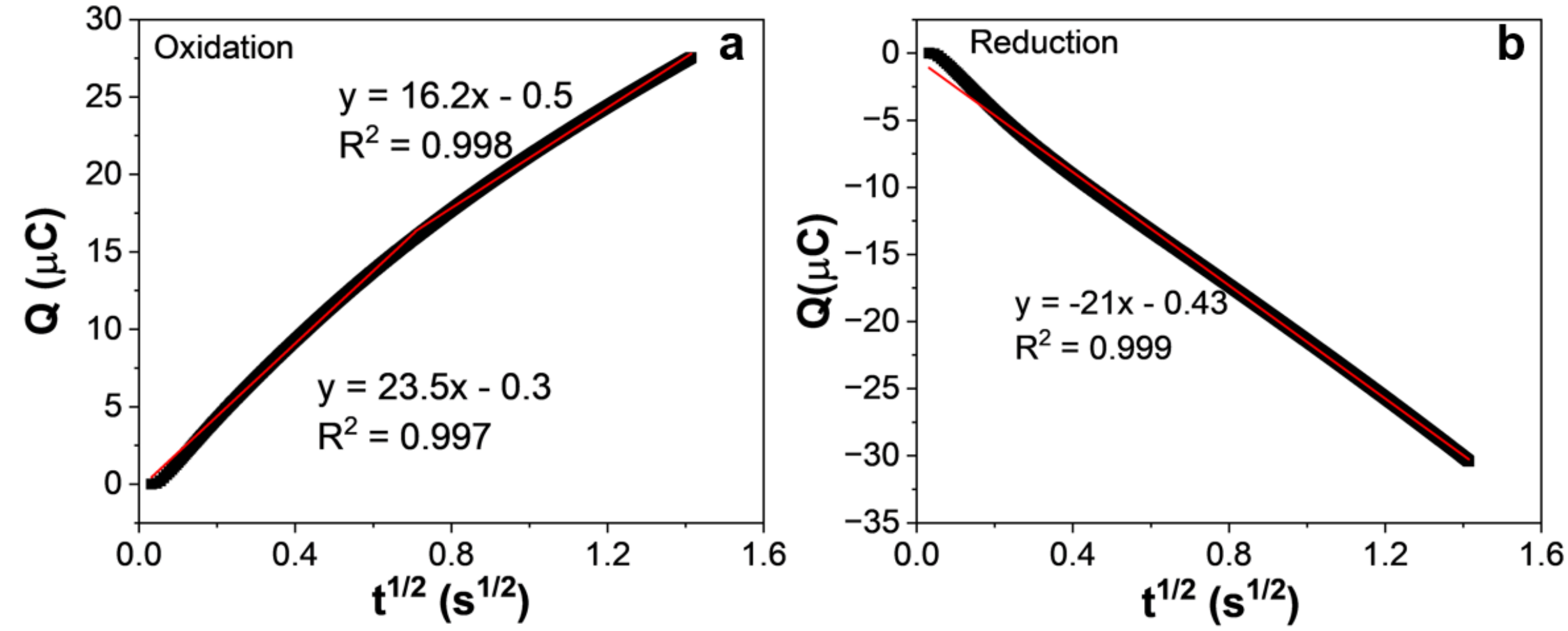

Figure S22. Anson plots from chronocoulometry of PEPP at 1/4c* in 0.1 M $TBAPF_6$ in DMF. (a) Oxidation (voltage applied –1.5 V vs Ag/Ag^+), in which two linear regions were observed. The early-time linear region corresponds to dissolved polymer, while the later-time region arises from aggregated or adsorbed polymer species. All D_{app} values were calculated from the first linear region. (b) Reduction in which one linear region was observed (voltage applied –2.5 V vs Ag/Ag+).

Table S1. Calculated apparent diffusion coefficients (D_{app}), homogenous($k_{ex,app}$) and heterogenous rate(k^0) for PEPP in this study.

Redox moiety	Molecular weight (kDa)	Conc. (mM)	D_{app} (cm^2/s)	$k_{ex,app}$ ($M^{-1}s^{-1}$)	k^0 (cm/s)
PEPP	41	4.67	$3.1 \pm 1.7\times 10^{-7}$ (O)<br>$2.0 \pm 1.4 \times 10^{-7}$(R)	$8.5 \pm 4.8 \times 10^{8}$ (O)<br>$5.5 \pm 4.1 \times 10^{8}$ (R)	$2.7 \pm 2.6 \times 10^{-5}$ (O)<br>$2.6 \pm 2.2 \times 10^{-5}$ (R)
		5.6	$2.3 \pm 1.9\times 10^{-7}$(O)<br>$2.1 \pm 0.23 \times 10^{-7}$(R)	$5.5 \pm 4.3 \times 10^{8}$ (O)<br>$4.9 \pm 0.53 \times 10^{8}$ (R)	$1.3 \pm 0.75 \times 10^{-4}$ (O)<br>$1.2 \pm 0.42 \times 10^{-4}$ (R)
		7	$2.0 \pm 0.18 \times 10^{-7}$ (O)<br>$1.8 \pm 0.43 \times 10^{-8}$ (R)	$3.8 \pm 0.69 \times 10^{8}$ (O)<br>$3.4 \pm 0.81 \times 10^{8}$ (R)	$8.5 \pm 4.3 \times 10^{-5}$ (O)<br>$7.8 \pm 3.2 \times 10^{-5}$ (R)
		9.33	$2.7 \pm 2.1 \times 10^{-7}$ (O)<br>$2.2 \pm 0.87 \times 10^{-7}$ (R)	$3.9 \pm 2.9 \times 10^{8}$ (O)<br>$3.1 \pm 1.2 \times 10^{7}$ (R)	$1.6 \pm 0.56 \times 10^{-4}$ (O)<br>$1.3 \pm 0.28 \times 10^{-4}$ (R)
		14	$6.6 \pm 1.1 \times 10^{-8}$ (O)<br>$7.4 \pm 2.1 \times 10^{-8}$ (R)	$6.2 \pm 1.1 \times 10^{7}$ (O)<br>$6.9 \pm 0.19 \times 10^{7}$ (R)	$7.5 \pm 2.3 \times 10^{-5}$ (O)<br>$8.1 \pm 2.8 \times 10^{-5}$ (R)
		28	$4.3 \pm 2.7 \times 10^{-8}$ (O)<br>$4.3 \pm 2.1 \times 10^{-8}$ (R)	$2.1 \pm 1.3 \times 10^{7}$ (O)<br>$2.0 \pm 1.0 \times 10^{7}$ (R)	$5.1 \pm 3.9 \times 10^{-5}$ (O)<br>$5.3 \pm 3.7 \times 10^{-5}$ (R)
		56	$1.07 \pm 0.81 \times 10^{-8}$ (O)<br>$4.3 \pm 2.8\times 10^{-8}$ (R)	$2.5 \pm 1.9 \times 10^{6}$ (O)<br>$1.0 \pm 0.65 \times 10^{7}$ (R)	$2.4 \pm 1.8 \times 10^{-5}$ (O)<br>$3.5 \pm 0.85 \times 10^{-5}$ (R)
		84	$4.2 \pm 1.2 \times 10^{-8}$ (O)<br>$4.8 \pm 0.6 \times 10^{-8}$ (R)	$6.6 \pm 1.8 \times 10^{6}$ (O)<br>$7.5 \pm 0.9 \times 10^{6}$ (R)	$2.6 \pm 1.6 \times 10^{-5}$ (O)<br>$2.7 \pm 1.4 \times 10^{-5}$ (R)

Table S2. Calculated apparent diffusion coefficients (D_{app}), homogenous($k_{ex,app}$) and heterogenous rate(k^0) for PVBP in this study.

Redox moiety	Molecular weight (kDa)	Conc. (mM)	D_{app} (cm^2/s)	$k_{ex,app}$ ($M^{-1}s^{-1}$)	k^0 (cm/s)
PVBP	13.6	5	$1.4 \pm 0.32 \times 10^{-7}$ (O)<br>$2.7 \pm 0.61 \times 10^{-7}$ (R)	$3.6 \pm 0.83 \times 10^{8}$ (O)<br>$7.1 \pm 1.6 \times 10^{8}$(R)	$1.9 \pm 0.98\times 10^{-4}$ (O)<br>$2.6 \pm 0.26\times 10^{-4}$ (R)
		6	$2.1 \pm 1.2 \times 10^{-7}$ (O)<br>$2.0 \pm 1.1 \times 10^{-7}$ (R)	$4.6 \pm 2.7 \times 10^{8}$ (O)<br>$4.4 \pm 2.4 \times 10^{8}$ (R)	$2.9 \pm 1.8 \times 10^{-4}$ (O)<br>$2.6 \pm 0.62 \times 10^{-4}$(R)
		7.5	$1.7 \pm 0.50 \times 10^{-7}$ (O)<br>$2.2 \pm 0.64 \times 10^{-7}$ (R)	$3.0 \pm 0.88 \times 10^{8}$ (O)<br>$3.8 \pm 1.1 \times 10^{8}$(R)	$2.1 \pm 2.0 \times 10^{-4}$ (O)<br>$2.4 \pm 0.13 \times 10^{-4}$(R)
		10	$6.1 \pm 5.4 \times 10^{-7}$ (O)<br>$4.7 \pm 4.5 \times 10^{-7}$ (R)	$8.0 \pm 7.2 \times 10^{8}$ (O)<br>$6.1 \pm 5.9 \times 10^{8}$ (R)	$3.3 \pm 1.5 \times 10^{-4}$ (O)<br>$2.9 \pm 1.4 \times 10^{-4}$(R)
		15	$1.2 \pm 0.62 \times 10^{-7}$ (O)<br>$1.2 \pm 0.52 \times 10^{-7}$ (R)	$1.1 \pm 0.54 \times 10^{8}$ (O)<br>$1.1 \pm 0.45 \times 10^{8}$ (R)	$1.5 \pm 1.3 \times 10^{-4}$ (O)<br>$1.5 \pm 0.34 \times 10^{-4}$(R)
		30	$1.5 \pm 0.10 \times 10^{-7}$ (O)<br>$1.1 \pm 0.10 \times 10^{-7}$ (R)	$6.6 \pm 4.3 \times 10^{7}$ (O)<br>$4.9 \pm 4.4 \times 10^{7}$ (R)	$5.1 \pm 2.2 \times 10^{-5}$ (O)<br>$4.8 \pm 2.1 \times 10^{-5}$(R)
		60	$9.6 \pm 9.4 \times 10^{-8}$ (O)<br>$5.6 \pm 3.2 \times 10^{-8}$(R)	$2.2 \pm 2.1 \times 10^{7}$ (O)<br>$1.3 \pm 0.74 \times 10^{7}$ (R)	$6.6 \pm 2.6 \times 10^{-5}$ (O)<br>$5.5 \pm 0.87 \times 10^{-5}$(R)
		90	$7.9 \pm 5.2 \times 10^{-8}$ (O)<br>$7.8 \pm 3.2 \times 10^{-8}$(R)	$1.1 \pm 0.76 \times 10^{7}$ (O)<br>$1.1 \pm 0.79 \times 10^{7}$ (R)	$2.2 \pm 1.9 \times 10^{-5}$ (O)<br>$2.1 \pm 1.6 \times 10^{-5}$(R)

Table S3. Calculated apparent diffusion coefficients (D_{app}), homogenous($k_{ex,app}$) and heterogenous rate(k^0) for PMAP in this study.

Redox moiety	Molecular weight (kDa)	Conc. (mM)	D_{app} (cm^2/s)	$k_{ex,app}$ ($M^{-1}s^{-1}$)	k^0 (cm/s)
PMAP	20.1	3.75	$2.6 \pm 0.39 \times 10^{-7}$ (O)<br>$3.5 \pm 0.84 \times 10^{-7}$ (R)	$1.1 \pm 0.16 \times 10^{9}$ (O)<br>$1.4 \pm 0.34 \times 10^{9}$ (R)	$3.3 \pm 2.2 \times 10^{-4}$ (O)<br>$4.1 \pm 3.5 \times 10^{-4}$(R)
		4.5	$2.7 \pm 2.6 \times 10^{-7}$ (O)<br>$2.3 \pm 0.5 \times 10^{-7}$(R)	$7.9 \pm 7.7 \times 10^{8}$ (O)<br>$6.6 \pm 1.5 \times 10^{7}$(R)	$1.8 \pm 0.62 \times 10^{-4}$ (O)<br>$1.8 \pm 0.11 \times 10^{-4}$(R)
		5.625	$1.1 \pm 0.47 \times 10^{-7}$ (O)<br>$4.3 \pm 0.28 \times 10^{-7}$(R)	$2.5 \pm 1.1 \times 10^{8}$ (O)<br>$2.9 \pm 0.65 \times 10^{8}$ (R)	$1.3 \pm 0.14 \times 10^{-4}$ (O)<br>$1.4 \pm 0.22 \times 10^{-4}$(R)
		7.5	$2.5 \pm 1.7 \times 10^{-7}$ (O)<br>$2.7 \pm 2.0 \times 10^{-7}$(R)	$4.4 \pm 3.0 \times 10^{8}$ (O)<br>$4.8 \pm 2.6 \times 10^{8}$ (R)	$1.4 \pm 0.58 \times 10^{-4}$ (O)<br>$1.5 \pm 0.47 \times 10^{-4}$ (R)
		11.25	$1.3 \pm 0.18 \times 10^{-7}$ (O)<br>$1.1 \pm 0.60 \times 10^{-7}$(R)	$1.5 \pm 0.21 \times 10^{8}$ (O)<br>$1.3 \pm 0.7 \times 10^{8}$ (R)	$9.8 \pm 1.5 \times 10^{-5}$ (O)<br>$9.1 \pm 3.3 \times 10^{-5}$ (R)
		22.5	$1.3 \pm 0.18 \times 10^{-7}$ (O)<br>$1.1 \pm 0.60 \times 10^{-7}$(R)	$9.1 \pm 3.0 \times 10^{7}$ (O)<br>$1.1 \pm 0.26 \times 10^{7}$ (R)	$4.7 \pm 1.5 \times 10^{-5}$ (O)<br>$5.1 \pm 1.9 \times 10^{-5}$ (R)
		45	$1.2 \pm 0.49 \times 10^{-7}$ (O)<br>$1.2 \pm 0.37 \times 10^{-7}$(R)	$3.5 \pm 1.4 \times 10^{7}$ (O)<br>$3.5 \pm 1.1 \times 10^{7}$ (R)	$1.6 \pm 1.4 \times 10^{-5}$ (O)<br>$1.7 \pm 1.5 \times 10^{-5}$ (R)
		67.5	$8.6 \pm 5.6 \times 10^{-8}$ (O)<br>$9.4 \pm 6.9 \times 10^{-8}$(R)	$2.1 \pm 1.2 \times 10^{7}$ (O)<br>$1.8 \pm 1.3 \times 10^{7}$ (R)	$1.8 \pm 2.3 \times 10^{-5}$ (O)<br>$1.9 \pm 2.4 \times 10^{-5}$ (R)[a]

Footnote *a*: *Large relative standard deviation is attributed to exponential error propagation inherent to the Nicholson framework at peak separations exceeding 250 mV.*

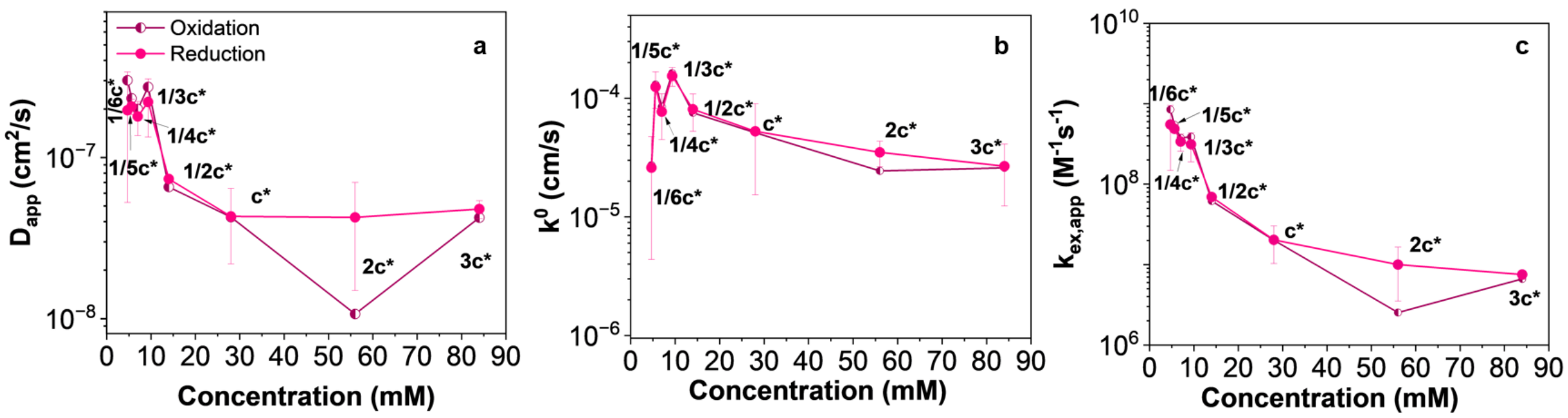

Figure S23. Kinetic parameters (for oxidation and reduction) of PEPP from chronocoulometry at different concentrations: a) D_{app}, b) $k_{ex,app}$, and c) k^0.

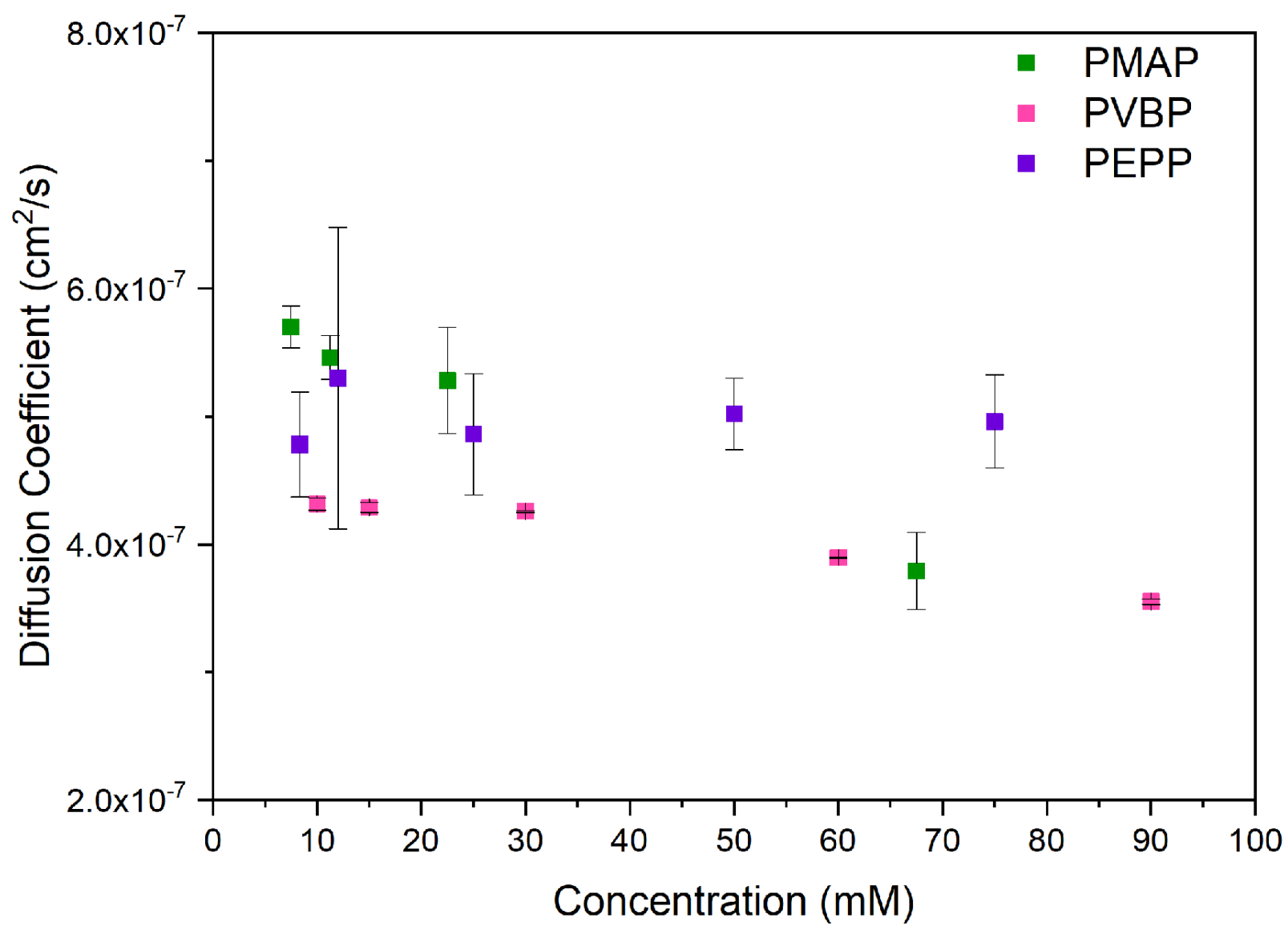

Figure S24. Diffusion coefficients of PMAP, PVBP, and PEPP obtained using dynamic light scattering.

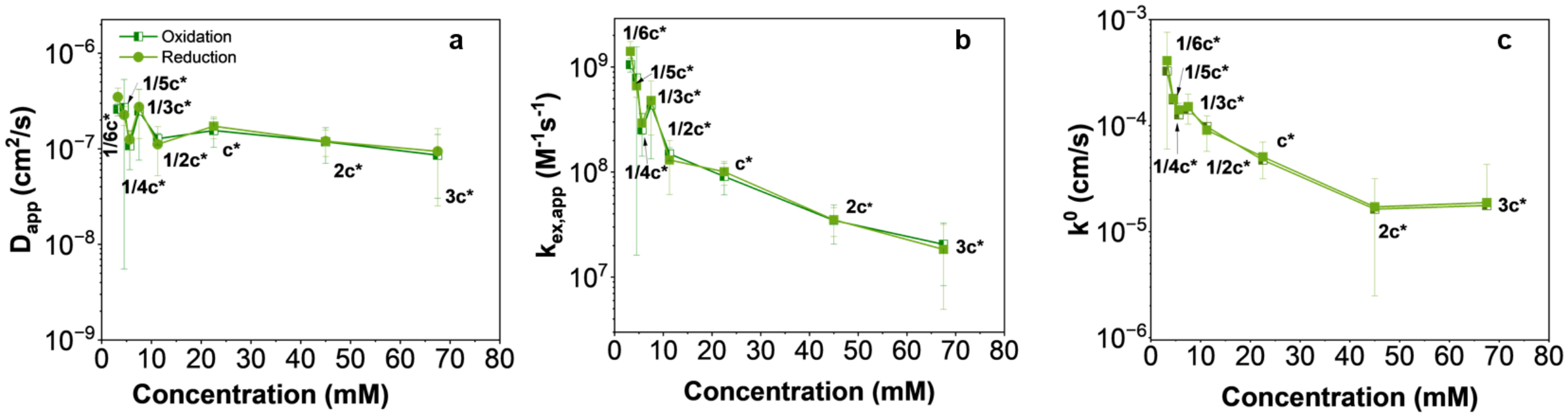

Figure S25. Kinetic parameters (for oxidation and reduction) of PMAP from chronocoulometry at different concentrations: a) D_{app}, b) $k_{ex,app}$, and c) k^0.

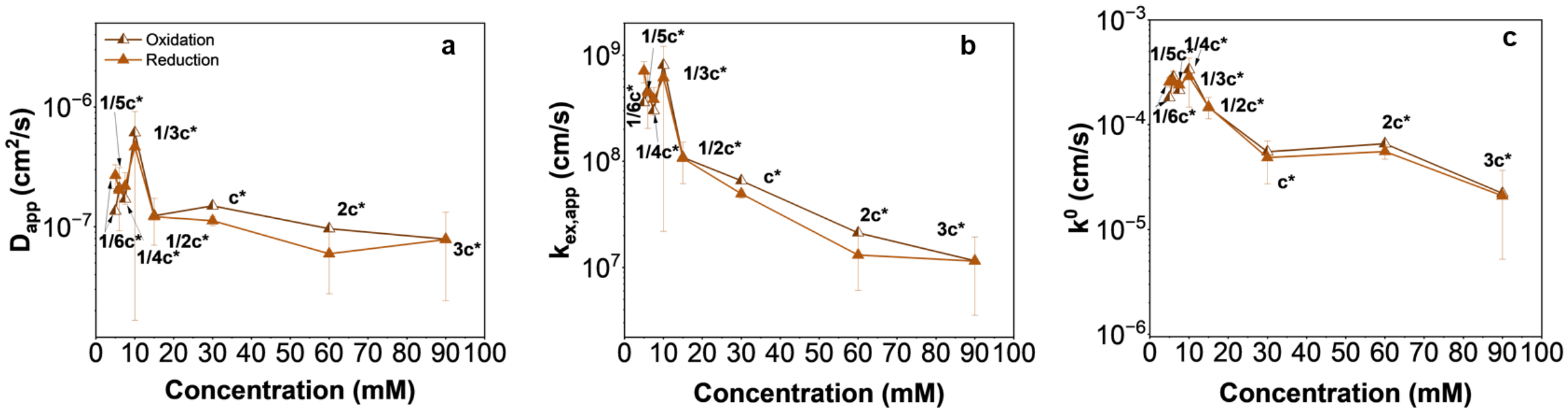

Figure S26. Kinetic parameters (for oxidation and reduction) of PVBP from chronocoulometry at different concentrations: a) $\underline{D}_{\mathrm{app}}$, b) $k_{\mathrm{ex,app}}$, and c) k^0.

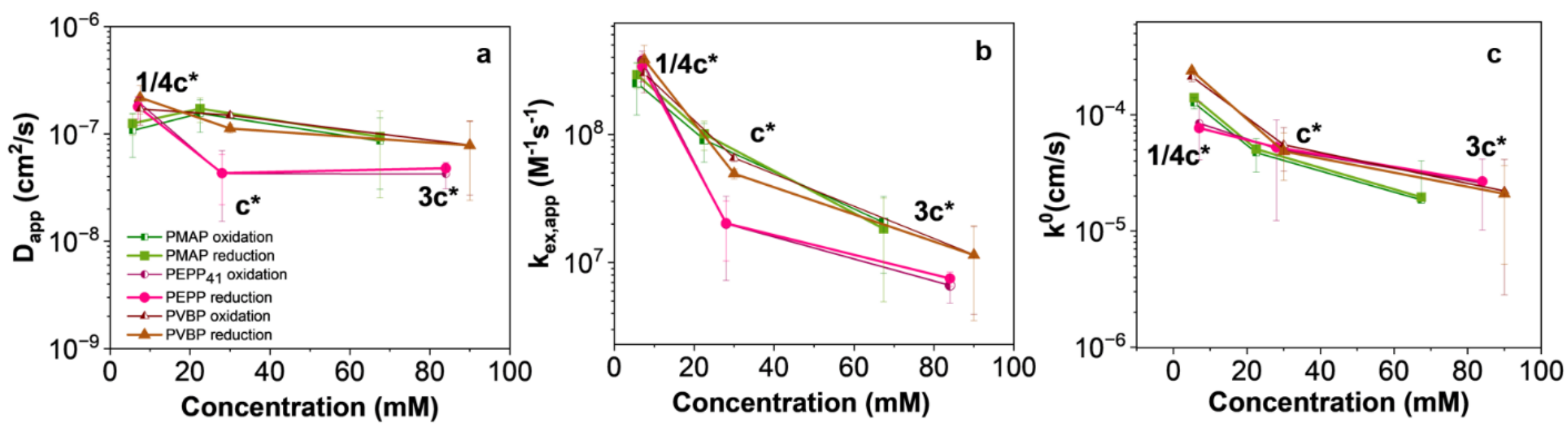

Figure S27. Kinetic parameters (for oxidation and reduction) of PEPP, PMAP, and PVBP from chronoamperometry at different concentrations: a) D_{app}, b) $k_{\mathrm{ex,app}}$, and c) k^0.

Table S4. Typically reported apparent diffusion coefficients (D_{app}), homogenous($k_{ex,app}$) and heterogenous rate(k^0) constants for of small molecules and polymers in solutions.

**Redox moiety**	**MW (g/mol)**	**Conc. (mM)**	**Electrolyte**	***D*_app (cm²/s)**	***k*_ex,app (M⁻¹s⁻¹)**	***k*⁰ (cm/s)**	**Ref.**
TEMPO-based polymer (P1)	20.2 x 10^3	2.5	0.1M NaCl in water	7.0 ± 0.5 ×10^{-8}	-	4.5 ± 0.1 ×10^{-5}	1
Viologen-based polymer (P2)	30.9 x 10^3	2.5	0.1M NaCl in water	7.6 ± 0.9×10^{-7}	-	9 ± 2 ×10^{-5}	1
P(TMA-*r*-SMA)	90 x 10^3	2	0.1M NaCl in water	3.6 x 10^{-6} (RS)<br>7.8 x 10^{-6} (RDE)	-	1.3 x 10^{-3}	2
PVMA	11 x 10^3	2	0.1M NaCl in water	1.6 x 10^{-6} (RS)<br>5.1x 10^{-6} (RDE)	-	2 x 10^{-3}	2
poly *o*-benzene dimer	432 x 10^3	10	0.1 M $LiBF_4$ in ACN	3.2 x 10^{-7}	6.7 x 10^8	1.1 ×10^{-3} ± 5 ×10^{-4}	3
poly *m*-benzene dimer	286 x 10^3	10	0.1 M $LiBF_4$ in ACN	4.1 x 10^{-7}	6.0 x 10^7	1.7 ×10^{-3} ± 5 ×10^{-4}	3
poly benzene monoviologen	53 x 10^3	10	0.1 M $LiBF_4$ in ACN		8.1 x 10^6	1.0 ×10^{-2} ± 5 ×10^{-4} (SECM)	3, 4
poly(benzyl ethyl viologen)	21-x 233 10^3	5	$TBAPF_6$ in ACN	1.7- 9.0 x 10^{-6} (RDE)<br>6.3-12 x 10^{-7} (SECM)	-	1.0-4.0 x 10^{-3} (RDE)<br>4.4-23 x 10^{-3} (SECM)	4
poly(benzyl ethyl viologen)	318 x 10^3	5	$TBAPF_6$ in ACN	1.2 x 10^{-5} (RDE)<br>6.6 x 10^{-7} (SECM)	1.5 x 10^7	3.0 ×10^{-3} RDE)<br>1.0 ×10^{-2} (SECM)	4, 5
Poly(aminoferrocene)	271 x 10^3	5	$TBAPF_6$ in ACN	-	2 x 10^5 and 3 x 10^6	-	4, 5
NMeph	161.2	Eutectic electrolyte	DME/DCE/LiTFSI<br>DMF/DCE/LiTFSI	4.8 × 10^{-6}<br>1.6 ×10^{-6}	-<br>-	-<br>-	6
N-ferrocenylphthalimide	332.2	100	1 M $TBABF_4$ in 1-3, dioxolane	3.21 x 10^{-6} (O)<br>3.65 x 10^{-6} (R)	-	3.14 x 10^{-4}(O)<br>3.37 x 10^{-4} (R)	7
Chloro- substituted phthalimide	181.1	100	1 M KPF_6 in acetonitrile	1.45 x 10^{-7} (O)<br>1.48 x 10^{-7} (R)	-	4.52 x 10^{-3}	7
Methyl-substituted phthalimide	161.1	100	1 M KPF_6 in acetonitrile	1.65 x 10^{-7} (O)<br>1.0 x 10^{-7} (R)	-	4.01 x 10^{-3}	8
Ethyl-substituted phthalimide	175.1	100	1 M KPF_6 in acetonitrile	1.71 x 10^{-7} (O)<br>1.74 x 10^{-7} (R)	-	3.84 x 10^{-3}	8
NO_2-substituted phthalimide	192.1	100	0.1 M KPF_6 in acetonitrile	2.43 x 10^{-7} (O)<br>2.6 x 10^{-7} (R)	-	3.51 x 10^{-3}	8
tert-Butyl-substituted phthalimide	203.1	100	0.1 M KPF_6 in acetonitrile	2.65 x 10^{-7} (O)<br>2.75 x 10^{-7} (R)	-	3.12 x 10^{-3}	8

Section S1: Estimation of the Electron-Hopping Contribution D_e

To justify the approximation $D_{app} \approx D_{phys}$ used in the main text, we compare the relative magnitudes of the electron hopping contribution, D_e, and the translational contribution, D_{phys}

The hopping contribution is computed in two steps as described in our previous paper.[9] First, the hopping rate k_{hop} estimated from Marcus–Hush theory:[10-12]

$$k_{hop} = \sqrt{\frac{\pi}{\lambda k_B T}} \frac{|V_{ij}|^2}{\hbar} exp\, exp \left(-\frac{(\Delta G_O+\lambda)^2}{4\lambda k_{BT}}\right)$$

In this expression, V_{ij} is the electronic coupling between redox sites, l is the reorganization energy, ΔG_0 is the charge-hopping reaction free energy, T is the temperature, k_B is Boltzmann's constant, and $\hbar$ is the reduced Planck constant. For symmetric hopping, we set $\Delta G_0 = 0$. The electronic couplings V_{ij} were obtained from our published[8] machine-learning model trained on data evaluating SOMO-LUMO interactions of phthalimide pairs, with ωB97X-D/def2-SV(P) level of theory (**Figure S28**). The total reorganization energy was taken as the sum of inner-sphere and outer-sphere contributions $\lambda = \lambda_{in} + \lambda_{out}$. The inner-sphere contribution was estimated using Nelsen's four-point method:[13]

$$\lambda_{in} = E_{neu}^{red} + E_{red}^{neu} - E_{neu}^{neu} - E_{red}^{red}$$

Where E_α^β denotes the single-point energy of a molecule in electronic state α evaluated at the geometry optimized for state β. All calculations were performed using Gaussian 16.3 [14] at the ωB97X-D/def2-SV(P)level of theory, with implicit DMF solvent described using the polarizable continuum model.

The outer-sphere contribution was estimated using Marcus's model:[10]

$$\lambda_{out} = (\Delta e)^2 \left(\frac{1}{2r_D} + \frac{1}{2r_A} + \frac{1}{R_{DA}}\right)\left(\frac{1}{n^2} - \frac{1}{\epsilon_r}\right)$$

where Δ_e is the transferred charge, r_D and r_A are the effective donor and acceptor radii, R_{DA} is the donor-acceptor separation, n is the refractive index, and ε_r is the static dielectric constant of the surrounding medium. In the dilute and semi-dilute solution-state systems, the polymer volume fraction is negligible compared with the solvent, so the dielectric response of the medium was approximated by that of DMF. Accordingly, we used $\varepsilon_{DMF} = 38.25$ and $n_{DMF} = 1.4305$ to parameterize solvent polarization in the outer reorganization-energy calculations.[15] Other parameters can be found in our previous paper.[9]

Following this, we use the random-walk expression:

$$D_e = \frac{k_{hop}\delta^2}{6}$$

to estimate the electron-hopping contribution to the apparent diffusion coefficient, where δ is the center-to-center hopping distance between redox sites (**Figure S29**). Overall, the estimated electron-hopping contribution D_e is on the order of 10^{-9} cm^2/s for all systems (**Figure S29**), whereas the physical diffusion coefficient D_{phys} is on the order of 10^{-6} cm^2/s as shown in the main text.

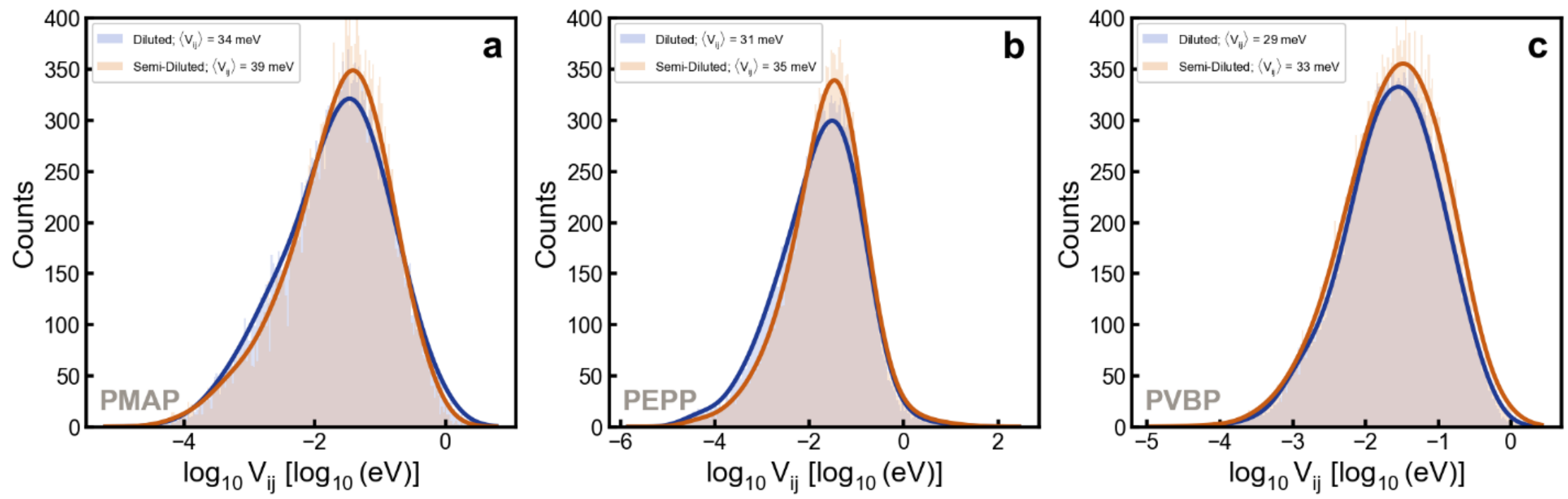

Figure S28. Electron Couplings Distribution for (a) PMAP, (b) PEPP, and (c) PVBP in dilute and semi-diluted conditions, as obtained from our Machine Learning Model.

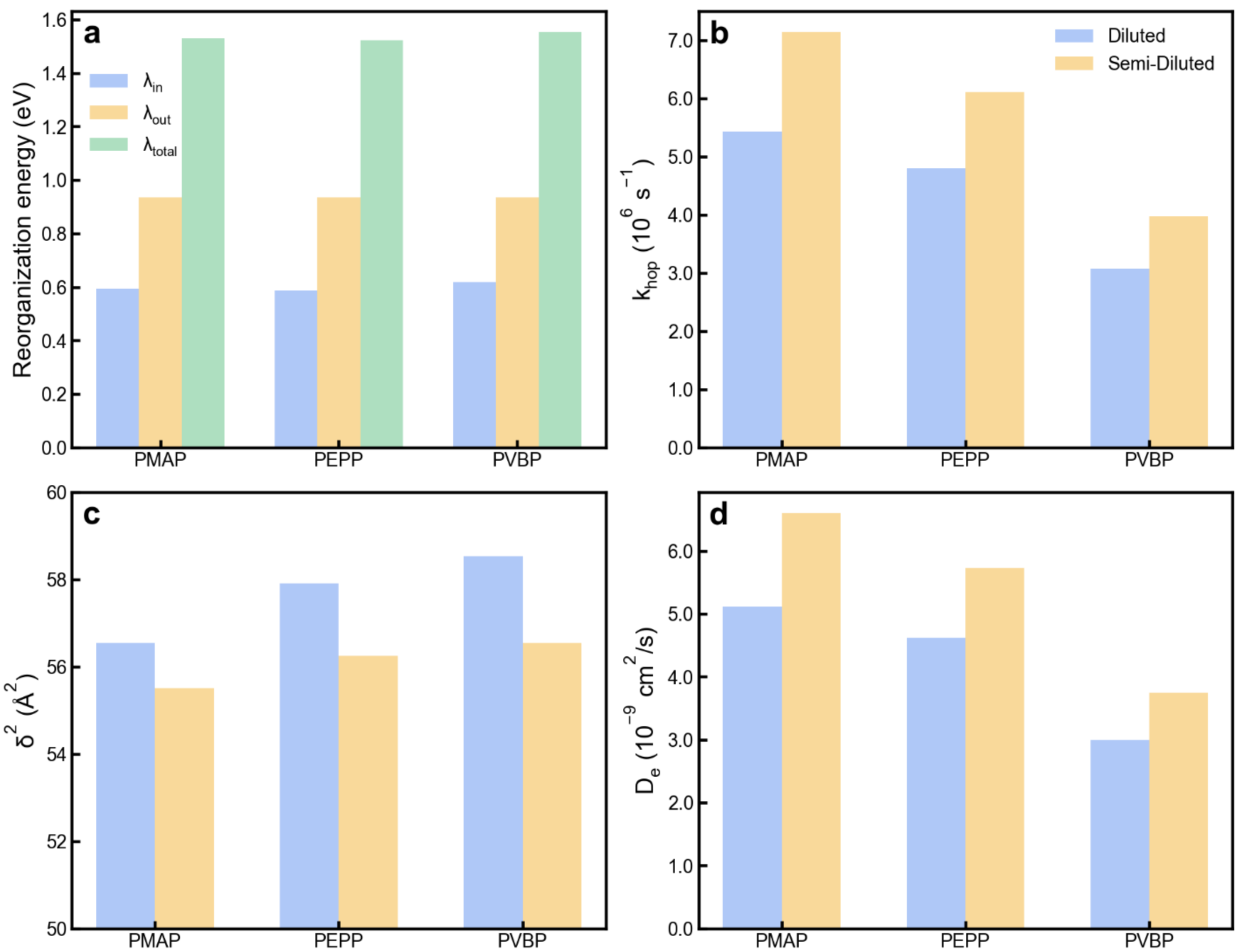

Figure S29. Predicted electron-transport parameters for the investigated redox-active polymers. (a) Reorganization energies, including λ_{in}, λ_{out}, λ_{total} .(b) Electron self-exchange rate constants, k_{hop}.(c) Average squared center-to-center hopping distances, δ^2.(d) Corresponding electron-hopping contributions to the apparent diffusion coefficient, D_e

References

(1) Janoschka, T.; Martin, N.; Martin, U.; Friebe, C.; Morgenstern, S.; Hiller, H.; Hager, M. D.; Schubert, U. S. An aqueous, polymer-based redox-flow battery using non-corrosive, safe, and low-cost materials. *Nature* **2015**, *527* (7576), 78-81. DOI: 10.1038/nature15746.
(2) Hatakeyama-Sato, K.; Igarashi, Y.; Oyaizu, K. Charge-transport kinetics of dissolved redox-active polymers for rational design of flow batteries. *RSC Advances* **2023**, *13* (1), 547-557. DOI: 10.1039/d2ra07208d (acccessed 7/3/2026).
(3) Burgess, M.; Chénard, E.; Hernández-Burgos, K.; Nagarjuna, G.; Assary, R. S.; Hui, J.; Moore, J. S.; Rodríguez-López, J. Impact of backbone tether length and structure on the electrochemical performance of viologen redox active polymers. *Chemistry of Materials* **2016**, *28* (20), 7362-7374.
(4) Burgess, M.; Moore, J. S.; Rodríguez-López, J. Redox Active Polymers as Soluble Nanomaterials for Energy Storage. *Accounts of Chemical Research* **2016**, *49* (11), 2649-2657. DOI: 10.1021/acs.accounts.6b00341.
(5) Burgess, M.; Hernández-Burgos, K.; Schuh, J. K.; Davila, J.; Montoto, E. C.; Ewoldt, R. H.; Rodríguez-López, J. Modulation of the Electrochemical Reactivity of Solubilized Redox Active Polymers via Polyelectrolyte Dynamics. *Journal of the American Chemical Society* **2018**, *140* (6), 2093-2104. DOI: 10.1021/jacs.7b08353.
(6) Zhang, C.; Niu, Z.; Ding, Y.; Zhang, L.; Zhou, Y.; Guo, X.; Zhang, X.; Zhao, Y.; Yu, G. Highly concentrated phthalimide-based anolytes for organic redox flow batteries with enhanced reversibility. *Chem* **2018**, *4* (12), 2814-2825.
(7) Hwang, S.; Kim, H.-s.; Ryu, J. H.; Oh, S. M. N-ferrocenylphthalimide; A single redox couple formed by attaching a ferrocene moiety to phthalimide for non-aqueous flow batteries. *Journal of Power Sources* **2018**, *395*, 60-65. DOI: https://doi.org/10.1016/j.jpowsour.2018.05.053.
(8) Chola, N. M.; Nagarale, R. K. Evaluation and degradation mechanism of phthalimide derivatives as anolytes for non-aqueous organic static batteries. *New Journal of Chemistry* **2022**, *46* (47), 22593-22601. DOI: 10.1039/d2nj03495f (acccessed 7/6/2026).
(9) Alessandri, R.; Li, C.-H.; Keating, S.; Mohanty, K. T.; Peng, A.; Lutkenhaus, J. L.; Rowan, S. J.; Tabor, D. P.; de Pablo, J. J. Structural, Ionic, and Electronic Properties of Solid-State Phthalimide-Containing Polymers for All-Organic Batteries. *JACS Au* **2024**, *4* (6), 2300-2311.
(10) Marcus, R. A. ON THE THEORY OF OXIDATION—REDUCTION REACTIONS INVOLVING ELECTRON TRANSFER. V. COMPARISON AND PROPERTIES OF ELECTROCHEMICAL AND CHEMICAL RATE CONSTANTS1. *The Journal of Physical Chemistry* **1963**, *67* (4), 853-857. DOI: 10.1021/j100798a033.
(11) Marcus, R. A.; Sutin, N. Electron transfers in chemistry and biology. *Biochimica et Biophysica Acta (BBA) - Reviews on Bioenergetics* **1985**, *811* (3), 265-322. DOI: https://doi.org/10.1016/0304-4173(85)90014-X.
(12) Hush, N. S. Electron transfer in retrospect and prospect: 1: Adiabatic electrode processes. *Journal of Electroanalytical Chemistry* **1999**, *460* (1), 5-29. DOI: https://doi.org/10.1016/S0022-0728(98)00371-4.
(13) Nelsen, S. F.; Blackstock, S. C.; Kim, Y. Estimation of inner shell Marcus terms for amino nitrogen compounds by molecular orbital calculations. *Journal of the American Chemical Society* **1987**, *109* (3), 677-682. DOI: 10.1021/ja00237a007.
(14) *Gaussian 16 Rev. C.01*; Wallingford, CT, 2016. (accessed.
(15) Haynes, W. M. *CRC Handbook of Chemistry and Physics*; CRC Press, 2016.
